# Superior anion induced shuttling behaviour exhibited by a halogen bonding two station rotaxane†
†Electronic supplementary information (ESI) available. CCDC 1454515. For ESI and crystallographic data in CIF or other electronic format see DOI: 10.1039/c6sc00783j


**DOI:** 10.1039/c6sc00783j

**Published:** 2016-04-28

**Authors:** Timothy A. Barendt, Sean W. Robinson, Paul D. Beer

**Affiliations:** a Chemistry Research Laboratory , Department of Chemistry , University of Oxford , 12, Mansfield Road , Oxford , OX1 3TA , UK . Email: paul.beer@chem.ox.ac.uk ; Fax: +44 (0)1865 272690 ; Tel: +44 (0)1865 285142

## Abstract

Two bistable halogen and hydrogen bonding-naphthalene diimide [2]rotaxanes have been prepared and the system incorporating a halogen bond donor anion recognition site is demonstrated to exhibit superior anion induced translational motion of the macrocyclic wheel component relative to the hydrogen bonding analogue. Proton NMR spectroscopy is used to estimate the percentage occupancies of the macrocycle at the respective station and importantly indicates that the halogen bonding rotaxane displays superior positional integrity in competitive protic solvent media as a consequence of strong halogen bond–halide anion binding interactions.

## Abstract 2

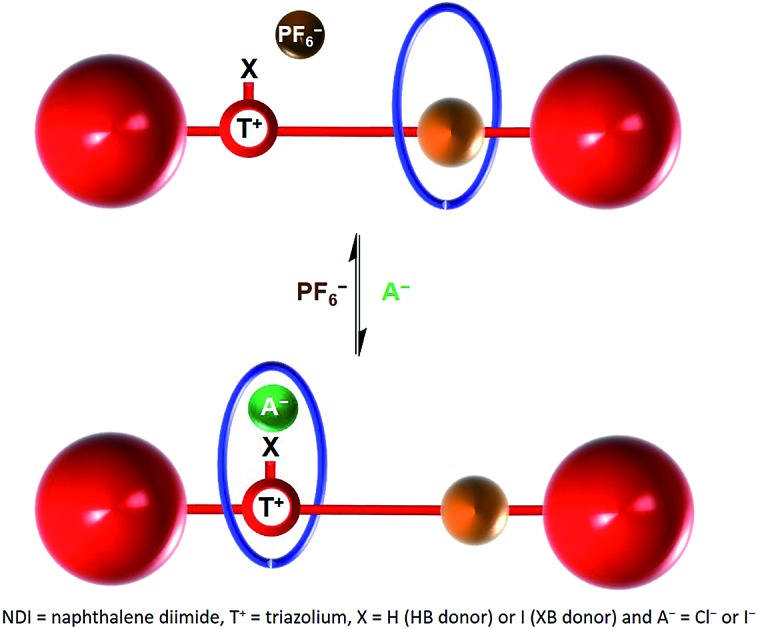
Two bistable halogen and hydrogen bonding-naphthalene diimide [2]rotaxanes have been prepared and the system incorporating a halogen bond donor anion recognition site is demonstrated to exhibit superior anion induced translational motion of the macrocyclic wheel component relative to the hydrogen bonding analogue.

## Introduction

With the growing demand for smaller and more efficient computing devices, it has been proposed that the next wave of technology could use individual molecules as a way of rapidly handling vast amounts of data on the nanometre scale.^1^ As a consequence, the controlling of molecular motion by using dynamic interlocked structures is receiving an increasing amount of interest.^2^–^7^ This is motivated by the promise of their potential applications in nanotechnology as machines,^7^ switches,^4^,^7^,^8^ sensors^9^,^10^ or delivery systems^11^–^14^ where dynamic behaviour is driven by a variety of light-, chemical- and redox-based stimuli.^4^,^7^,^15^–^18^ Whilst examples of cation binding-induced co-conformational change^4^,^7^ within a rotaxane or catenane are prevalent in the literature, systems that use anions^19^–^27^ as an external stimulus to produce molecular motion are relatively rare.

According to IUPAC “a halogen bond [XB] occurs when there is evidence of a net attractive interaction between an electrophilic region associated with a halogen atom in a molecular entity and a nucleophilic region in another, or the same, molecular entity”.^28^ The integration of XB donor motifs into the scaffold of an anion receptor has resulted in contrasting and, in some cases, enhanced anion recognition behaviour in comparison to hydrogen bonding (HB) receptor analogues in protic organic solvent mixtures and recently in pure water.^29^–^32^ This has also led to the development of the first interlocked structure utilising XB anion recognition to control translational molecular motion.^33^

**Fig. 1:**
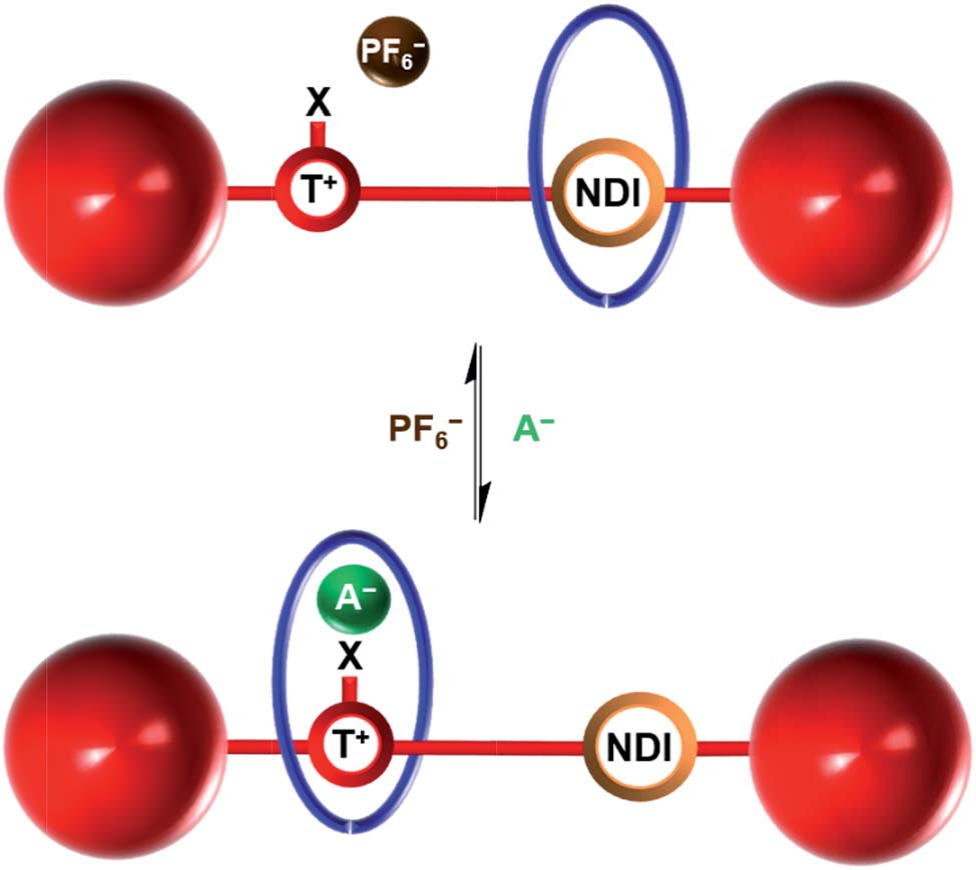
Schematic of the anion induced translational molecular motion within a bistable [2]rotaxane. NDI = naphthalene diimide, T^+^ = triazolium, X = H (HB donor) or I (XB donor) and A^–^ = Cl^–^ or I^–^.

Herein we report the syntheses of two novel cationic XB and HB bistable rotaxanes and demonstrate their capability to perform molecular shuttling upon changing the nature of the counter anion and the solvent. Both rotaxanes comprise of an isophthalamide-containing macrocycle threaded by a potential two station axle. The axle component contains an electron-deficient naphthalene diimide (NDI) motif capable of aromatic donor–acceptor charge-transfer interactions with the electron-rich hydroquinone groups of the macrocycle^34^,^35^ and as the second station, an XB iodo- or HB proto-triazolium group capable of anion coordination. Molecular motion of the macrocyclic wheel component from the NDI station to the triazolium group is demonstrated upon addition of a coordinating halide anion as the external stimulus (Fig. 1). Importantly, superior shuttling behaviour is exhibited by the XB rotaxane in competitive CDCl_3_ : CD_3_OD solvent mixtures as a consequence of strong XB–halide anion binding.

## Results and discussion

### Synthesis of HB and XB two station rotaxanes

**Scheme 1:**
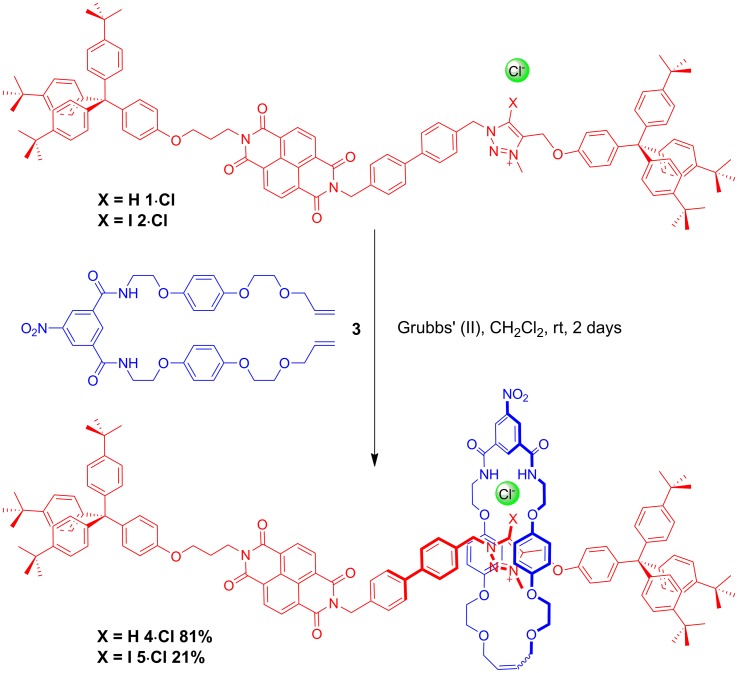
Syntheses of HB and XB rotaxanes **4·Cl** and **5·Cl** by an anion-templated clipping methodology.

The HB and XB two station rotaxanes **4·Cl** and **5·Cl** were prepared by multistep synthetic pathways as described in the ESI.† Chloride anion-templation was used to facilitate ring closing metathesis (RCM) of a bis-vinyl-functionalised macrocyclic precursor **3** around the appropriate HB or XB asymmetric axle (**1·Cl** and **2·Cl** respectively) using Grubbs' II catalyst in dry dichloromethane. Preparative silica thin-layer chromatography purification afforded the desired rotaxanes (Scheme 1) which were characterised by ^1^H, ^13^C and ^1^H 2D ROESY NMR spectroscopy and by high resolution mass spectrometry (see ESI†).

### Investigations into anion-induced molecular motion

**Fig. 2:**
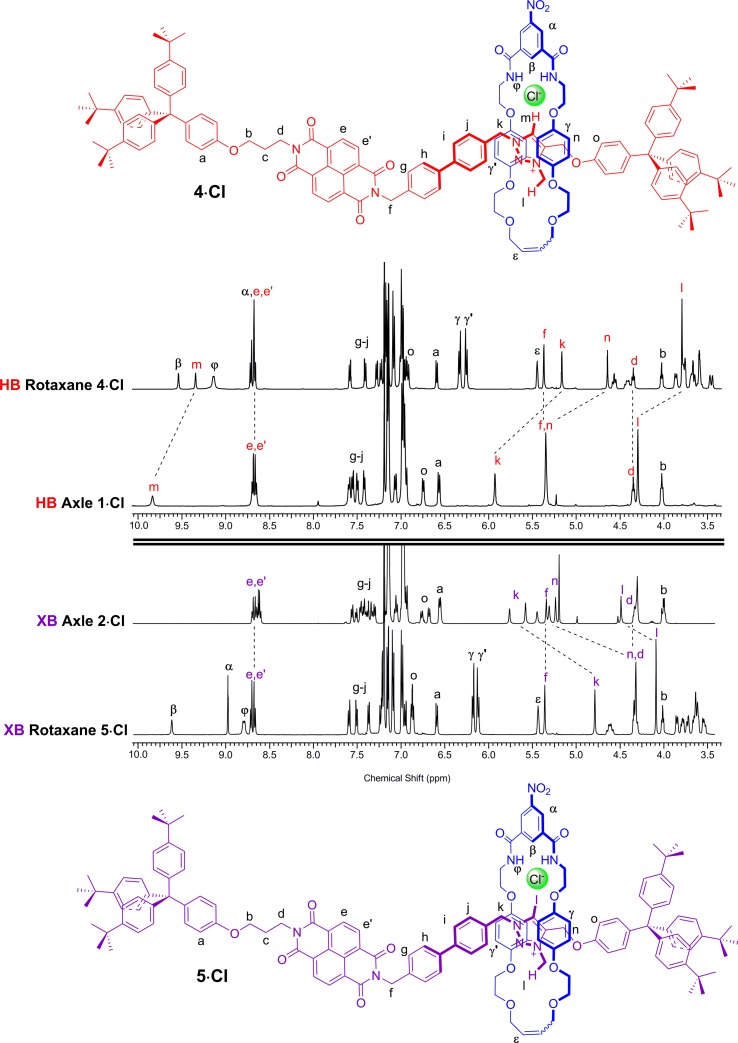
Comparison of the truncated ^1^H NMR spectra (CDCl_3_, 298 K, 400 MHz) of axles **1·Cl** and **2·Cl** and [2]rotaxanes **4·Cl** and **5·Cl** with their respective expected co-conformations.

A comparison of the ^1^H NMR spectra of the axle components **1·Cl** and **2·Cl** with their respective rotaxanes **4·Cl** and **5·Cl**, in CDCl_3_, was used to indicate the co-conformation of both interlocked structures in the presence of the coordinating chloride anion (Fig. 2). Exclusive upfield shifts of the triazolium proton H_m_ (in the case of the HB rotaxane **4·Cl**), *N*-methyl triazolium group H_l_ and the alkyl groups (H_k_ and H_n_) either side of the triazolium station are observed on comparing the ^1^H NMR spectra of the axle and rotaxane. This is indicative of halide anion coordination by multiple HB and, in the case of **5·Cl**, XB, interactions within the unique three-dimensional cavity created between the axle and the macrocycle occupying the triazolium station. In contrast there is no change in the chemical shift of the NDI aromatic protons H_e,e′_ or its proximal alkyl protons H_f_ and H_d_ which suggests the macrocycle resides almost exclusively at the triazolium station in both rotaxanes. These co-conformations were confirmed by two-dimensional ^1^H ROESY NMR experiments in CDCl_3_ that showed, amongst other evidence, only cross coupling between hydroquinone protons of the macrocycle H_γ,γ′_ and triazolium *N*-methyl protons H_l_, with no interaction between H_γ,γ′_ and the NDI protons H_e,e′_ (see ESI†).

**Fig. 3:**
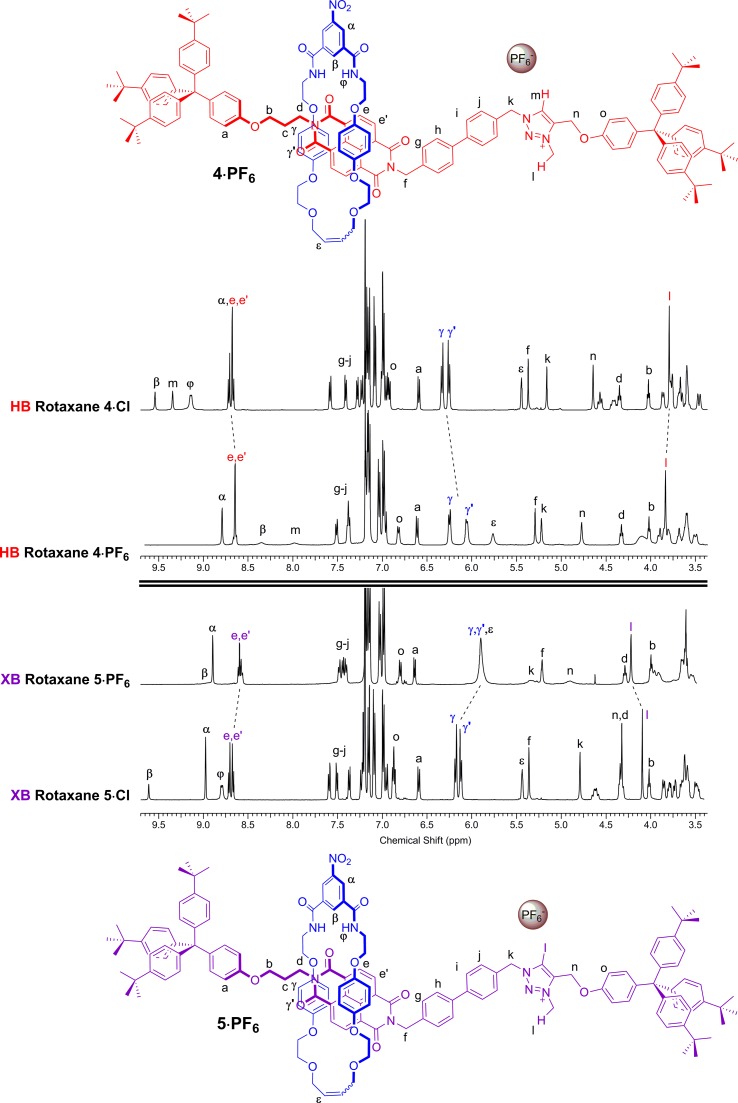
Comparison of the truncated ^1^H NMR spectra (CDCl_3_, 298 K, 400 MHz) of [2]rotaxanes **4·Cl**, **5·Cl**, **4·PF_6_** and **5·PF_6_** with their predicted co-conformations in the presence of PF_6_^–^.

Anion exchange to the corresponding hexafluorophosphate salts was achieved by repeatedly passing a solution of the rotaxane through a column containing an Amberlite® IRA-400 resin loaded with PF_6_^–^. A comparison of the resulting ^1^H NMR spectra of rotaxanes **4·PF_6_** and **5·PF_6_** with their respective chloride salts **4·Cl** and **5·Cl** in CDCl_3_ indicated a change in co-conformation had occurred as a result of shuttling of the macrocycle component to occupy the NDI station of the axle (Fig. 3). This is inferred by upfield shifts of the macrocycle hydroquinone protons H_γ,γ′_ which are indicative of stronger aromatic donor–acceptor charge-transfer interactions between the axle's electron-deficient NDI motif and macrocycle hydroquinone groups. In addition, upfield shifts of the NDI station protons H_e,e′_, in contrast to a downfield shift of the *N*-methyl triazolium protons H_l_, also suggests that shuttling of the macrocyclic component to the NDI station had taken place. Interestingly, the largest shifts are observed in the XB two station rotaxane **5·PF_6_** indicating a greater degree of translocation within this system has occurred than the HB analogue **4·PF_6_**. The changes to the co-conformations are further evidenced by the ^1^H 2D ROESY NMR spectra of **4·PF_6_** and **5·PF_6_**, both showing the appearance of new correlations between resonance signals arising from the macrocycle hydroquinone protons H_γ,γ′_ and the NDI protons H_e,e′_ (see ESI†).

**Fig. 4:**
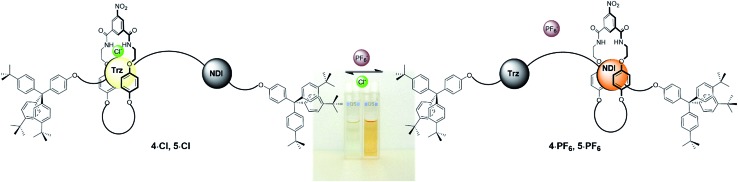
Schematic of the anion-induced molecular shuttling exhibited by rotaxanes **4·A** and **5·A** and the colour of their two co-conformations in CDCl_3_ solution.

Originally colourless as chloride salts **4·Cl** and **5·Cl**, donor–acceptor charge-transfer interactions between the macrocycle hydroquinone motifs and the extended π surface of the NDI station resulting from the shuttling behaviour upon conversion to rotaxanes **4·PF_6_** and **5·PF_6_** gave rise to a bright, distinctive orange colour (Fig. 4). Hence molecular motion can be detected by the naked eye through this colorimetric sensing response and was also demonstrated to be reversible using UV-vis spectroscopic studies (see ESI†).

### X-ray crystallography^36^

**Fig. 5:**
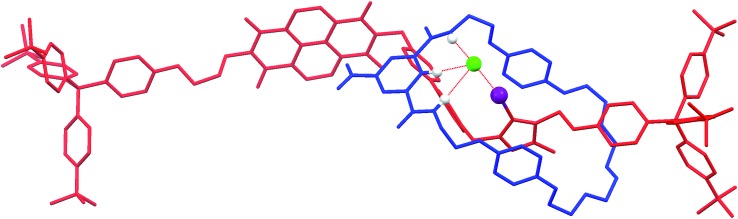
X-ray crystal structure of **5·Cl** showing the macrocycle (blue) situated at the iodo-triazolium station on the axle (red), templated by the coordinated chloride anion (green). Notable hydrogen and halogen bonding interactions are shown as dotted red lines with the atoms involved shown as spheres. Non-coordinating hydrogens and the co-crystallised chloroform and water molecules are omitted for clarity.

Crystals suitable for X-ray diffraction structural analysis were obtained for the chloride salt of the XB rotaxane **5·Cl** by slow evaporation of a solution of the rotaxane in 1 : 1 CDCl_3_ : CD_3_OD. The rotaxane crystallises in *P*1[combining macron] where the asymmetric unit comprises the rotaxane as a 1 : 1 complex with chloride, as well as one molecule each of chloroform and water. Notably, the solid-state structure of the chloride salt of the XB rotaxane shows the macrocycle situated exclusively at the triazolium station, templated by the presence of the coordinating chloride anion (*D*_I···Cl^–^_ = 2.987(6) Å) (Fig. 5). This chloride anion···iodo-triazolium halogen bond is much shorter than the sum of the van der Waals radii (80%), indicating a very strong interaction. The structure is in agreement with the aforementioned ^1^H NMR evidence for the favoured co-conformation of the molecular shuttle in the presence of a coordinating anion.

### Effect of anion and solvent variation on percentage occupancies

**Fig. 6:**
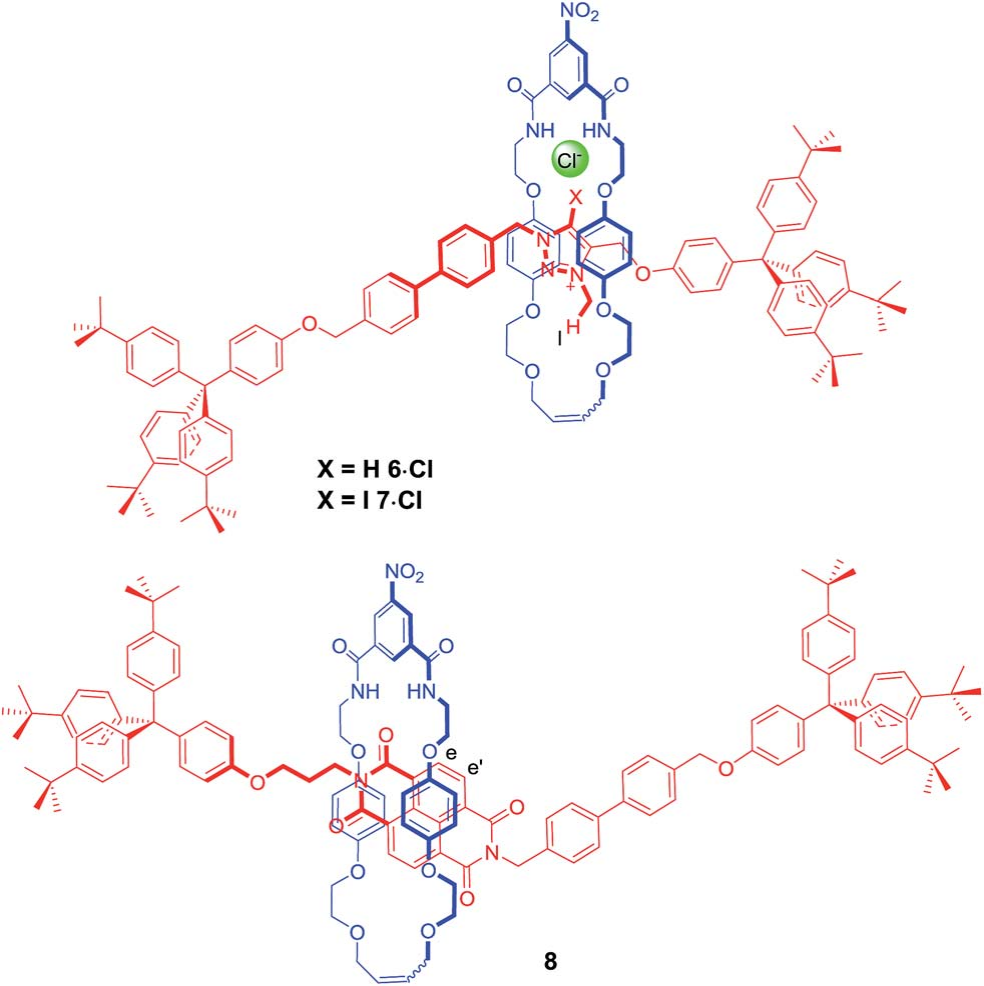
The structures of the three model one station rotaxanes **6·Cl**, **7·Cl** and **8**.

In order to further investigate how the effect of varying the nature of the counter anion influenced the co-conformation of the XB and HB molecular shuttles, three new one-station rotaxanes containing either a proto-triazolium (**6·Cl**), iodo-triazolium (**7·Cl**) or NDI (**8**) motif were synthesised by multistep synthetic pathways and characterised by ^1^H and ^13^C NMR spectroscopy and by high resolution mass spectrometry as described in the ESI† (Fig. 6). These rotaxanes acted as models for the two station shuttles and enabled estimations of the station percentage occupancies by the macrocycle to be determined in the presence of non-coordinating PF_6_^–^ and chloride and iodide anions.^37^

The ^1^H NMR spectra of these three rotaxanes in CDCl_3_ gave the proton chemical shift values of each station's diagnostic protons when there was complete occupancy by the macrocycle; specifically the *N*-methyl protons of the triazolium ring (H_l_) and those of the NDI aromatic protons (H_e,e′_). The chemical shift values for the same protons at 0% occupancy were obtained from the ^1^H NMR spectra in CDCl_3_ of the two-station axles **1·A** and **2·A** for a particular anion (A). Using these data, chemical shift scales were determined with respect to each station in the presence of each anion, to characterise the two extremes of macrocycle occupation (see ESI†). This method was necessary because the dynamic behaviour of the bistable rotaxanes was fast on the ^1^H NMR timescale.

**Fig. 7:**
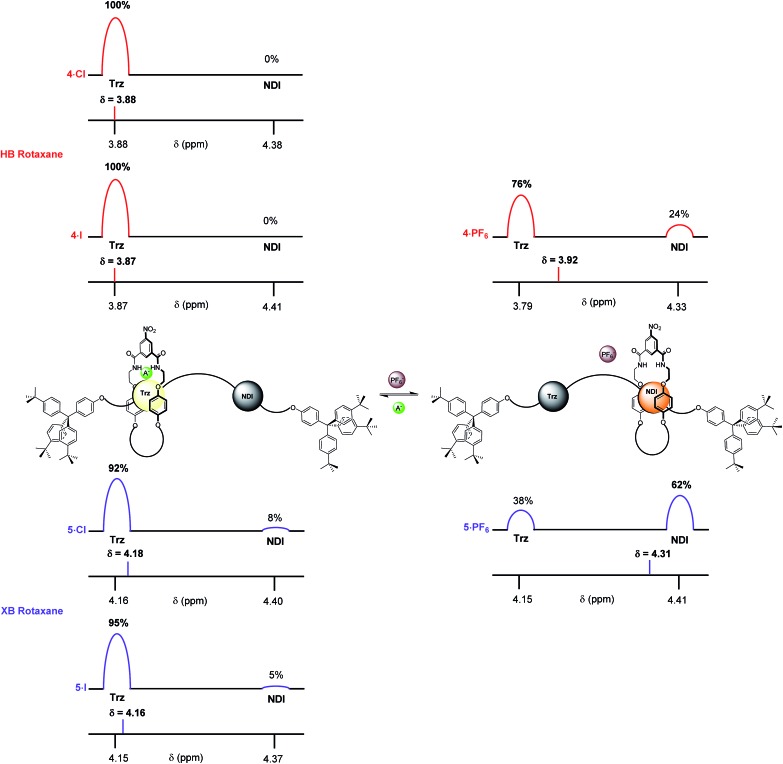
The estimated percentage occupancies (based upon H_l_ protons) of the HB and XB rotaxanes **4·A** and **5·A** as chloride, iodide and PF_6_^–^ salts as determined by ^1^H NMR spectroscopy in CDCl_3_ solution. A^–^ = Cl^–^ or I^–^ and Trz = triazolium.

The complete occupation of the triazolium station by the macrocycle in the HB two station rotaxane system in the chloride salt (**4·Cl**) was confirmed by comparison of the chemical shift of the *N*-methyl triazolium protons in **4·Cl** (*δ*(H_l_) = 3.88 ppm) with the *δ* HB *N*-methyl triazolium chloride chemical shift scale. This was supported by an analogous comparison of the chemical shift of the NDI protons, *δ*(H_e,e′_), in **4·Cl** with the NDI chemical shift scale which indicated zero occupancy of the NDI station. This method was used to determine the macrocycle occupancies of both HB and XB rotaxane **4·A** and **5·A** halide and PF_6_^–^ salts using the appropriate *N*-methyl triazolium chemical shift scale (reported in Fig. 7) and concurred using the NDI chemical shift scale (see ESI†).

From these data it may be seen that both two-station rotaxanes exhibit anion induced molecular motion in CDCl_3_ solution (Fig. 7). The preference for the triazolium station in the chloride salts (**4·Cl** and **5·Cl**) was evident for both HB (100% occupancy of the triazolium station) and XB (92%) rotaxane switches, giving excellent positional integrity within this state. The same was true for the iodide salts (**4·I** and **5·I**), with the HB and XB rotaxanes estimated to have 100% and 95% occupancies of their triazolium stations respectively. Upon exchange to the non-coordinating PF_6_^–^ salt, both systems demonstrated an increase in occupancy of the NDI station (Fig. 7), as expected from the one- and two-dimensional ^1^H NMR spectroscopy data discussed earlier. The largest occupation of the NDI station was found to occur with the XB rotaxane **5·PF_6_** (62%) relative to the HB rotaxane **4·PF_6_** (24%).^38^ This indicated the XB rotaxane behaves as a superior molecular shuttle to the HB analogue on account of the larger swing in percentage occupancy of the stations upon anion exchange (>54% for the XB rotaxane *vs.* 24% for the HB rotaxane).

The propensity for anion receptors containing XB donor groups to exhibit enhanced halide, in particular iodide, anion recognition properties over their HB analogues in protic organic solvents^30^,^31^ provided the impetus for investigating the shuttling behaviour in more competitive solvent mixtures. The percentage occupancies of the HB and XB rotaxane PF_6_^–^, chloride and iodide salt systems were estimated in competitive 4 : 1 CDCl_3_ : CD_3_OD and 1 : 1 CDCl_3_ : CD_3_OD solvent mixtures *via* analogous ^1^H NMR spectroscopic experiments monitoring the chemical shifts of the respective rotaxane salt *N*-methyl triazolium H_l_ protons. Importantly, the results revealed a significantly enhanced positional integrity, in favour of the triazolium station for the XB rotaxane halide salt systems.

**Fig. 8:**
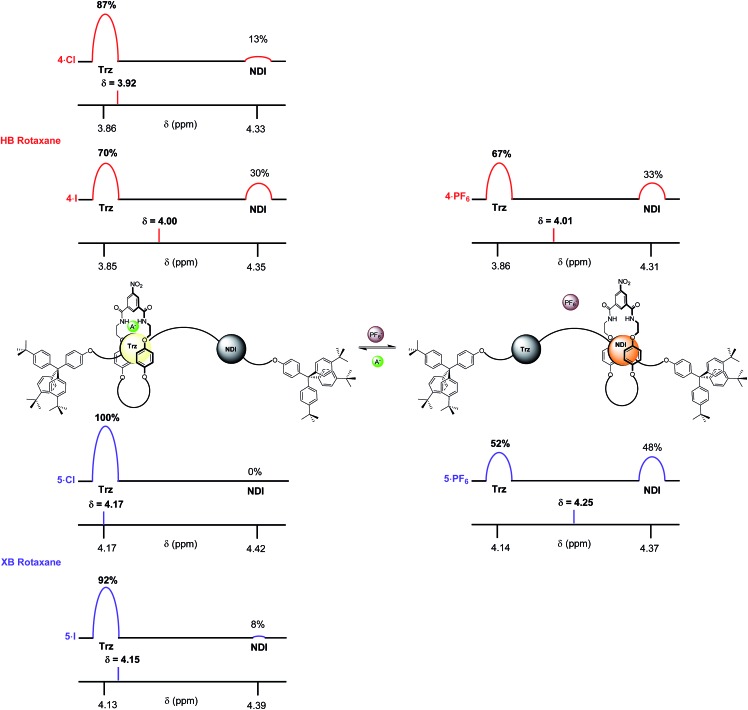
The estimated percentage occupancies (based upon H_l_ protons) of the HB and XB rotaxanes **4·A** and **5·A** as chloride, iodide and PF_6_^–^ salts as determined by ^1^H NMR spectroscopy in 4 : 1 CDCl_3_ : CD_3_OD solution. A^–^ = Cl^–^ or I^–^ and Trz = triazolium.

In 4 : 1 CDCl_3_ : CD_3_OD the difference in behaviour between XB and HB rotaxanes was most strongly manifested within the iodide salt of the rotaxanes. XB rotaxane **5·I** exhibited a 92% occupancy of the triazolium station whereas the HB analogue **4·I** was estimated at 70% (Fig. 8). The differences between the chloride salt occupancies of the triazolium stations of the two rotaxanes were, by contrast, smaller: XB rotaxane **5·Cl** was 100% *vs.* 87% for the HB rotaxane **4·Cl** (Fig. 8). This is a consequence of superior halide affinity afforded by strong XB formation from the rotaxane's iodo-triazolium anion recognition site in comparison to the weaker proto-triazolium HB donor motif in the competitive solvent medium. Upon exchange to their respective PF_6_^–^ salts, an enhanced positional integrity in favour of the NDI station was observed within the XB system **5·PF_6_** (48% occupancy of the NDI station), relative to the HB rotaxane **4·PF_6_** (33%), the same trend as found in CDCl_3_ solution.

**Fig. 9:**
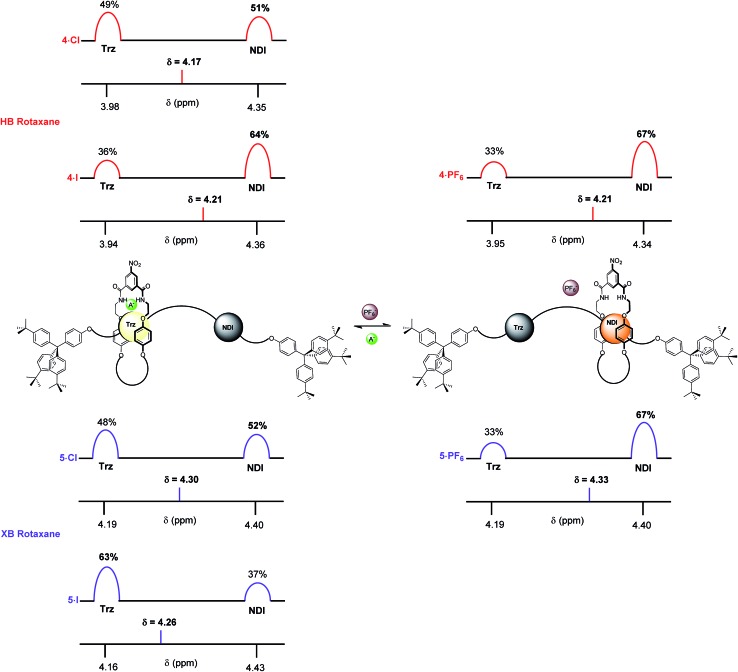
The estimated percentage occupancies (based upon H_l_ protons) of the HB and XB rotaxanes **4·A** and **5·A** as iodide and PF_6_^–^ salts as determined by ^1^H NMR spectroscopy in 1 : 1 CDCl_3_ : CD_3_OD solution. A^–^ = Cl^–^ or I^–^ and Trz = triazolium.

Finally, the percentage occupancies of both XB and HB rotaxane iodide and PF_6_^–^ salts were estimated in the more competitive solvent medium of 1 : 1 CDCl_3_ : CD_3_OD using ^1^H NMR spectroscopy experiments based upon the H_l_ protons (Fig. 9). The HB and XB rotaxanes were estimated to have the same occupancy of the NDI station as their PF_6_^–^ salts (67%). However, a significantly larger preference for the triazolium station was exhibited in the iodide salt by the XB system **5·I** (63% occupancy of the triazolium station), over the HB system **4·I** (36%). This enabled the XB shuttle to still perform effectively in 1 : 1 CDCl_3_ : CD_3_OD and produce a significant percentage of iodide induced molecular motion whereas the HB system afforded only negligible changes in the occupancies of the stations upon anion exchange. Analogous to previous solvent systems, there were smaller differences between occupancies of the triazolium stations in the chloride salts of HB and XB rotaxanes **4·Cl** and **5·Cl** (Fig. 9).

These experiments demonstrate that an enhanced positional integrity for the triazolium station in the halide state is achievable upon increasing the competitive nature of the solvent system, but only when it is facilitated by the XB interaction with iodide. As such, the XB rotaxane switch is demonstrated to be a superior example of a synthetic molecular shuttle over its HB analogue in both CDCl_3_ and competitive CDCl_3_ : CD_3_OD solvent mixtures.

## Conclusions

In conclusion, the synthesis of five novel rotaxanes including two bistable rotaxanes and three model mono station systems are reported. Both cationic XB/HB two station rotaxanes were demonstrated to undergo halide anion induced molecular switching of their co-conformations in CDCl_3_ solution, with the added benefit that this shuttling behaviour resulted in a distinct colour change of the solution, giving a naked eye response to the translocation of the macrocyclic component. The XB two station rotaxane **5** demonstrated superior molecular shuttling in CDCl_3_ solution over the HB analogue rotaxane **4**. Upon increasing the competitive nature of the solvent mixture to 4 : 1 and 1 : 1 CDCl_3_ : CD_3_OD, the XB two station rotaxane again demonstrated superior molecular shuttling. This was driven by the XB interaction between iodide and the iodo-triazolium XB donor anion recognition site within the axle component. These results were reflected by determined estimations of the percentage occupancies of the stations using ^1^H NMR spectroscopy. This is the first example of using XB in concert with donor–acceptor charge-transfer interactions to exert significant control over solution phase molecular shuttling motion within an interlocked supramolecular system. Importantly this highlights the real and exciting potential XB has in the future design of dynamic interlocked mechanically bonded nanotechnological switchable systems.

## Supplementary Material

Supplementary information

Crystal structure data
