# Supplementary material for: Superior anion induced shuttling behaviour exhibited by a halogen bonding two station rotaxane
Source: Chem Sci. 2016 Apr 28;7(8):5171–80. doi: 10.1039/c6sc00783j (PMC6020254; doi:10.1039/c6sc00783j)
Supplement: Supplementary file 1 [file SC-007-C6SC00783J-s001.pdf]

Electronic Supplementary Material (ESI) for Chemical Science.
This journal is © The Royal Society of Chemistry 2016

Supporting Information

Superior Anion Induced Shuttling Behaviour Exhibited by a Halogen Bonding Two Station Rotaxane

Timothy A. Barendt, Sean W. Robinson and Paul D. Beer

Chemistry Research Laboratory, Department of Chemistry University of Oxford, 12 Mansfield Road, Oxford, OX1 3TA

Email: paul.beer@chem.ox.ac.uk

Contents

1) **Part I: Synthesis** **S2**
2) **Part II: 2D ^{1}H NMR ROESY spectra** **S18**
3) **Part III: UV-Vis Spectroscopy** **S22**
4) **Part IV: Anion exchange molecular motion studies** **S23**
5) **Part V: X-ray Diffraction** **S34**

Part I: Synthesis

General Information

All commercial solvents and reagents were used as purchased, unless otherwise stated. Anhydrous solvents were degassed with N_2 and dried by passing them through an MBraun-800 column. Triethylamine was distilled and stored over KOH pellets. Grubbs' second generation catalyst and $Cu(MeCN)_4 \cdot PF_6$ were stored in a desiccator with P_2O_5. TBTA was prepared following a literature procedure.[1] Water was distilled and microfiltered using a Milli-Q Millipore machine. Chromatography was undertaken using silica gel (particle size: 40-63 µm) or preparative TLC plates (20 × 20 cm, 1 cm silica thickness).

Axle and rotaxane components were fully anion exchanged using a column containing an Amberlite® ion exchange resin that had been loaded with the desired anion. Amberlite® was "loaded" by washing the resin with $NaOH_{(aq)}$ (10%), H_2O, and either $NH_4Cl_{(aq)}$ (1 M), $NH_4I_{(aq)}$ (1 M) or $NH_4PF_{6(aq)}$ (0.1 M), followed by further H_2O, and the solvent (45:45:10 $CHCl_3$:MeOH:H_2O) to be used in the anion exchange. The compound was then dissolved in 5 – 10 mL of 45:45:10 $CHCl_3$:MeOH:H_2O and passed through the column at least three times to achieve complete anion exchange. After this the solvent was removed *in vacuo*, the residue redissolved in $CHCl_3$ (5 – 10 mL) and washed with H_2O (5 – 10 mL). After drying the organic phase over anhydrous $MgSO_4$ the solvent was removed *in vacuo* to give the product.

^{1}H, ^{13}C, ^{19}F and ^{31}P NMR spectra were recorded using Bruker AVIII400 and Bruker AVIII500 spectrometers. Mass spectra were recorded on a Waters LCT Premier (low resolution), Waters MALDI Micro MX or a Bruker µTOF instrument (high resolution).

The method for coupling amines with 1,4,5,8-naphthalenetetracarboxylic dianhydride to form NDI containing asymmetric axles **17**, **18** and **27** was adapted from a literature procedure.[2] The compounds **3**,[3] **9**,[4] **11**,[5] **16**[6] and **19**[7] were all synthesised according to literature procedures.

Synthesis of two-station rotaxanes 4·A and 5·A

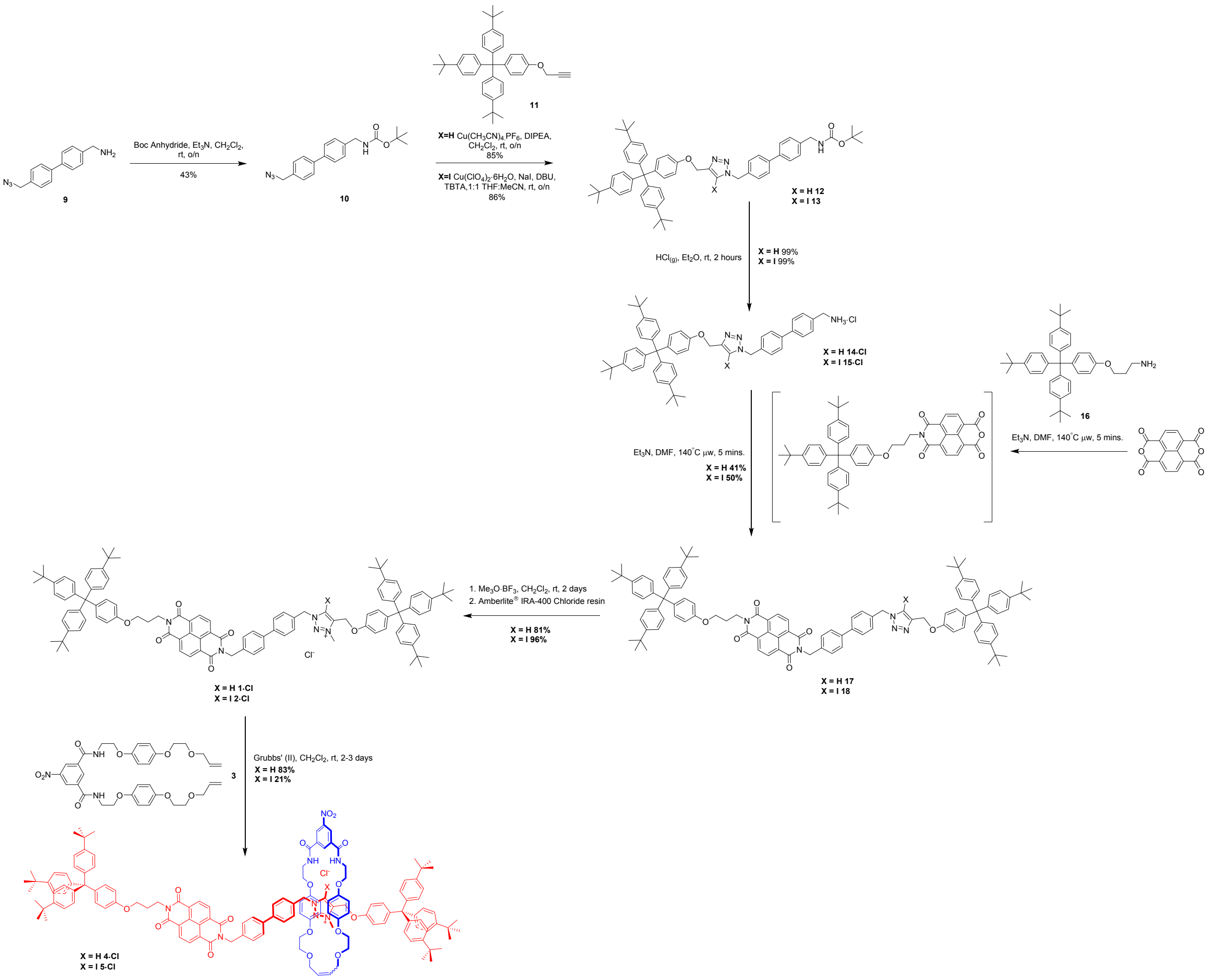

*Scheme S1. Synthetic route to two-station rotaxanes **4·Cl** and **5·Cl***

Biphenyl mono azide BOC-protected amine 10

Amine **9** (2.69 g, 0.01 mol, 1 eq.) was dissolved in dry CH_2Cl_2 (125 mL) and dry Et_3N (1.57 mL, 0.01 mmol, 1 eq.) was added. The solution was cooled to 0°C and Boc anhydride (4.91 g, 0.02 mmol, 2 eq.) in dry CH_2Cl_2 (125 mL) was added dropwise over five minutes. After addition, the reaction mixture was stirred at 0°C under $N_{2(g)}$ for 20 minutes, and the reaction mixture left to stir overnight at room temperature under $N_{2(g)}$. This solution was then diluted with CH_2Cl_2 (200 mL) and worked up by washing with a saturated $NaHCO_{3(aq)}$ solution (1 × 150 mL) and H_2O (1 × 150 mL). The organic phase was dried over anhydrous $MgSO_4$ and the solvent removed *in vacuo* to give the product **10** as an off-white solid (1.62 g, 4.80 mmol, 43%).

^{1}H NMR (400 MHz, $CDCl_3$) δ = 7.59 (4H, dd, J=14.79, 8.07 Hz, Ar*H*), 7.35 - 7.42 (4H, m, Ar*H*), 4.40 (2H, s, C*H*$_2$), 4.38 (2H, d, J=5.75 Hz, C*H*$_2$), 1.49 (9H, s, C*H*$_3$).

^{13}C NMR (101 MHz, $CDCl_3$) δ = 155.9, 140.8, 139.5, 138.3, 134.3, 128.6, 127.9, 127.4, 127.2, 79.5, 54.5, 44.3, 28.4

MS (ESI): m/z calc. for $C_{19}H_{22}N_4O_2$ $[M+Na]^+$: 361.16; found: 361.10.

Proto triazole BOC-protected amine axle pre-cursor 12

Azide **10** (19 mg, 0.06 mmol, 1 eq.) was dissolved in dry CH_2Cl_2 (2 mL). Alkyne functionalised terphenyl stopper compound **11** (30 mg, 0.06 mmol, 1 eq.), TBTA (6 mg, 0.01 mmol, 0.2 eq.), Cu(CH$_3$CN)4·PF6 (4 mg, 0.01 mmol, 0.2 eq.) and DIPEA (19 µL, 0.12 mmol, 2 eq.) were added and the resulting solution was left to stir overnight at room temperature. The reaction mixture was then washed with 25% $NH_4OH_{(aq)}$ (1 × 2 mL) and the organic phase was collected, dried over anhydrous $MgSO_4$ and the solvent removed *in vacuo*. The resulting residue was purified by silica gel column chromatography (99:1 CH_2Cl_2: MeOH) and compound **12** was isolated as a yellow solid (45 mg, 0.05 mmol, 85%).

^{1}H NMR (400 MHz, $CDCl_3$) δ = 7.66 (1H, s, triazole Ar*H*), 7.56 - 7.60 (2H, m, biphenyl Ar*H*), 7.53 (2H, d, *J* = 8.2 Hz, biphenyl Ar*H*), 7.33 - 7.39 (4H, m, biphenyl Ar*H*), 7.20 - 7.26 (6H, m, stopper Ar*H*), 7.04 - 7.12 (8H, m, stopper Ar*H*), 6.84 (2H, d, *J* = 8.9 Hz, stopper Ar*H*), 5.58 (2H, s, C*H*$_2$), 5.51 (2H, s, C*H*$_2$), 5.18 (2H, s, C*H*$_2$), 1.48 (9H, s, BOC C*H*$_3$), 1.30 (27H, s, stopper C*H*$_3$)

^{13}C NMR (101 MHz, $CDCl_3$) δ = 156.0, 148.3, 144.0, 141.3, 140.1, 139.1, 134.7, 133.4, 132.2, 130.6, 129.0, 128.6, 128.5, 127.9, 127.4, 127.2, 124.0, 123.6, 113.2, 63.0, 62.0, 54.0, 47.0, 34.2, 31.3, 30.8, 28.3

MS (ESI): m/z calc. for $C_{59}H_{69}N_4O_3$ $[M+H]^+$: 881.53; found: 881.61.

Iodo triazole BOC-protected amine axle pre-cursor 13

Azide **10** (100 mg, 0.3 mmol, 1 eq.) was dissolved in THF (2 mL). NaI (177 mg, 1.2 mmol, 4 eq.) and $Cu(ClO_4)_2{\cdot}6H_2O$ (219 mg, 0.6 mmol, 2 eq.) were added and the resulting suspension was left to stir for five minutes. TBTA (15.7 mg, 0.03 mmol, 0.1 eq.) and DBU (45 mg, 0.3 mmol, 1 eq.) were added to the mixture. Finally, alkyne functionalised terphenyl stopper compound **11** (160 mg, 0.3 mmol, 1 eq.), CH_3CN (2 mL) and CH_2Cl_2 (ten drops) were added and the reaction mixture was left to stir overnight in the dark. The mixture was then washed with 25% $NH_4OH_{(aq)}$ (1 × 3 mL) and the organic phase was collected, dried over anhydrous $MgSO_4$ and the solvent removed *in vacuo*. The resulting residue was purified by silica gel column chromatography (CH_2Cl_2). The iodo triazole containing compound **13** was isolated as an off-white solid (256 mg, 0.25 mmol, 86%).

^{1}H NMR (400 MHz, $CDCl_3$) δ ppm 7.54 (4H, dd, J=12.90, 8.13 Hz, biphenyl Ar*H*), 7.33 - 7.39 (4H, m, biphenyl Ar*H*), 7.23 (6H, d, J=8.68 Hz, stopper Ar*H*), 7.12 (8H, s, stopper Ar*H*), 6.91 (2H, d, J=8.93 Hz, stopper Ar*H*), 5.65 (2H, s, C*H*$_2$), 5.11 (2H, s, C*H*$_2$), 4.36 (2H, d, J=5.50 Hz, C*H*$_2$), 1.48 (9H, s, BOC C*H*$_3$), 1.31 (27H, s, stopper C*H*$_3$).

^{13}C NMR (101 MHz, $CDCl_3$) δ ppm 156.2, 148.3, 147.9, 144.0, 141.1, 140.3, 139.3, 138.4, 133.0, 132.3, 130.7, 129.1, 128.6, 128.4., 128.0, 127.5, 127.3, 124.0, 113.6, 68.0, 63.1, 61.8, 54.0, 34.3, 31.46, 28.4, 25.6.

MS (ESI): m/z calc. for $C_{59}H_{67}IN_4O_3$ $[M+H]^+$: 1007.43; found: 1007.43.

Proto triazole amine axle pre-cursor 14·Cl

12 (45 mg, 0.05 mmol) was dissolved in dry Et_2O (5 mL). $HCl_{(g)}$ was generated *in situ* by the dropwise addition of concentrated H_2SO_4 to $NaCl_{(s)}$ and bubbled through the solution for 25 minutes. After that the solution was stirred for two hours at room temperature. The resulting precipitate was filtered and washed with Et_2O to give an off white solid **14·Cl**, the hydrochloride salt of the free amine (41 mg, 0.05 mmol, 99%).

^{1}H NMR (400MHz, MeOD) δ = 7.94 (1H, s, triazole Ar*H*), 7.62 (4H, dd, *J* = 18.2 Hz, *J* = 8.1 Hz, biphenyl Ar*H*), 7.36 - 7.51 (4H, m, biphenyl Ar*H*), 7.18 - 7.25 (6H, m, stopper Ar*H*), 7.01 - 7.09 (8H, m, stopper Ar*H*), 6.83 (2H, d, *J* = 8.9 Hz, stopper Ar*H*), 5.58 (2H, s, C*H*$_2$), 5.13 (2H, s, C*H*$_2$), 4.06 (2H, s, C*H*$_2$), 1.23 (27H, s, stopper C*H*$_3$)

^{13}C NMR (126MHz, MeOD) δ = 155.7, 148.1, 143.8, 140.8, 140.4, 140.1, 133.6, 132.0, 130.4, 129.2, 128.5, 127.5, 127.4, 123.8, 112.9, 62.8, 61.4, 61.0, 54.1, 53.7, 44.6, 42.8, 33.9, 31.0, 29.4, 28.8, 18.1, 16.8

MS (ESI): m/z calc. for $C_{54}H_{61}N_4O$ $[M-Cl]^+$: 781.48; found: 781.51.

Iodo triazole amine axle pre-cursor 15·Cl

13 (298 mg, 0.30 mmol) was dissolved in dry Et_2O (25 mL). $HCl_{(g)}$ was generated *in situ* by the dropwise addition of concentrated $H_2SO_{4(aq)}$ to $NaCl_{(s)}$ and bubbled through the solution for 25 minutes. After that the solution was stirred for two hours at room temperature. The resulting precipitate was filtered and washed with Et_2O to give the hydrochloride salt of the product as a white solid **15·Cl** (280 mg, 0.30 mmol, 99%).

^{1}H NMR (400 MHz, MeOD) δ = 7.58 - 7.65 (2H, m, biphenyl Ar*H*), 7.53 - 7.58 (2H, m, biphenyl Ar*H*), 7.46 - 7.52 (2H, m, biphenyl Ar*H*), 7.28 - 7.39 (2H, m, biphenyl Ar*H*), 7.20 (6H, d, *J* = 8.7 Hz, stopper Ar*H*), 7.01 - 7.12 (8H, m, stopper Ar*H*), 6.79 - 6.91 (2H, m, stopper Ar*H*), 5.66 (2H, s, C*H*$_2$), 5.06 (2H, s, C*H*$_2$), 4.10 (2H, s, C*H*$_2$), 1.26 (27H, s, stopper C*H*$_3$)

^{13}C NMR (101MHz, MeOD) δ = 155.8, 148.1, 143.8, 140.8, 140.2, 140.1, 133.2, 132.0, 130.4, 129.2, 128.2, 127.4, 127.2, 123.8, 113.3, 62.8, 61.3, 53.7, 49.1, 48.9, 48.7, 48.5, 48.3, 48.1, 47.8, 42.7, 33.9, 31.0

MS (MALDI-TOF): *m/z* calc. for $C_{54}H_{61}ClIN_4O$ $[M + H]^+$: 945.36; found: 945.05.

Proto triazole NDI axle 17

The propylamine functionalised terphenyl stopper compound **16** (20 mg, 0.04 mmol, 1 eq.) and commerically available 1,4,5,8-naphthalenetetracarboxylic dianhydride (10 mg, 0.04 mmol, 1 eq.) were suspended in 1 mL of dry, degassed DMF with dry Et_3N (6 µL, 0.04 mmol, 1 eq.) in a sealed microwave vial. The suspension was sonicated for ten minutes before heating under microwave irradiation for five minutes at 140°C. The resulting yellow solution was allowed to cool and the vial opened to allow for the addition of amine **14·Cl** (29 mg,

0.04 mmol, 1 eq.) and further dry Et_3N (6 µL, 0.04 mmol, 1 eq.). The vial was resealed and the suspension sonicated for 30 minutes before heating under microwave irradiation for a further five minutes at 140°C. Upon cooling the yellow solution was placed immediately into a round bottom flask and reduced *in vacuo* to give a golden yellow residue. This crude reaction mixture was purified by silica gel column chromatography (99:1 CH_2Cl_2: MeOH) to yield the desired neutral axle compound **17** (23 mg, 0.02 mmol, 41%).

^{1}H NMR (400MHz, $CDCl_3$) δ = 8.69 - 8.81 (4H, m, NDI Ar*H*), 7.60 - 7.68 (2H, m, biphenyl Ar*H*), 7.47 - 7.59 (5H, m, biphenyl Ar*H* and triazole Ar*H*), 7.33 (2H, d, *J* = 8.2 Hz, biphenyl Ar*H*), 7.19 - 7.29 (12H, m, stopper Ar*H*), 6.98 - 7.14 (16H, m, stopper Ar*H*), 6.84 (2H, d, *J* = 8.9 Hz, stopper Ar*H*), 6.64 (2H, d, *J* = 8.9 Hz, stopper Ar*H*), 5.56 (2H, s, C*H*$_2$), 5.44 (2H, s, C*H*$_2$), 5.17 (2H, s, C*H*$_2$), 4.38 - 4.47 (2H, m, C*H*$_2$), 4.04 - 4.16 (2H, m, C*H*$_2$), 2.21 - 2.32 (2H, m, C*H*$_2$), 1.31 (54H, d, *J* = 3.4 Hz, stopper C*H*$_3$)

^{13}C NMR (101MHz, $CDCl_3$) δ = 162.9, 162.8, 156.5, 156.1, 148.3, 148.3, 144.9, 144.1, 144.0, 141.2, 140.2, 139.7, 139.6, 136.0, 133.5, 132.3, 132.1, 131.2, 131.0, 130.7, 129.7, 128.5, 127.7, 127.3, 126.7, 126.7, 126.5, 124.0, 122.5, 113.2, 112.9, 65.9, 63.0, 63.0, 62.0, 53.9, 43.7, 38.6, 34.3, 31.4, 27.9

MS (ESI): m/z calc. for $C_{108}H_{112}N_5O_6$ $[M+H]^+$: 1575.86; found: 1575.98.

Iodo triazole NDI axle 18

The propylamine functionalised terphenyl stopper compound **16** (40 mg, 0.07 mmol, 1 eq.) and commerically available 1,4,5,8-naphthalenetetracarboxylic dianhydride (19 mg, 0.07 mmol, 1 eq.) were suspended in 1 mL of dry, degassed DMF with dry Et_3N (11 µL, 0.07 mmol, 1 eq.) in a sealed microwave vial. The suspension was sonicated for ten minutes before heating under microwave irradiation for five minutes at 140°C. The resulting brown solution was allowed to cool and the vial opened to allow for the addition of amine **13·Cl** (76 mg, 0.07 mmol, 1 eq.) and further dry Et_3N (11 µL, 0.07 mmol, 1 eq.). The vial was resealed and the suspension sonicated for 30 minutes before heating under microwave irradiation for a further five minutes at 140°C. Upon cooling the brown solution was placed immediately into a round bottom flask and the solvent removed *in vacuo* to give a golden brown residue. This crude reaction mixture was purified by silica gel column chromatography (99:1 CH_2Cl_2: MeOH) to yield the desired neutral axle compound **18** (59 mg, 0.03 mmol, 50%).

^{1}H NMR (400MHz, $CDCl_3$) δ = 8.76 (4H, q, *J* = 7.9 Hz, NDI Ar*H*), 7.59 - 7.67 (2H, m, biphenyl Ar*H*), 7.47 - 7.56 (4H, m, biphenyl Ar*H*), 7.31 - 7.39 (2H, m, biphenyl Ar*H*), 7.23 (12H, d, *J* = 8.4 Hz, stopper Ar*H*), 6.98 - 7.13 (16H, m, stopper Ar*H*), 6.85 - 6.92 (2H, m, stopper Ar*H*), 6.64 (2H, d, *J* = 8.9 Hz, stopper Ar*H*), 5.62 (2H, s, C*H*$_2$), 5.43 (2H, s, C*H*$_2$), 5.09 (2H, s, C*H*$_2$), 4.43 (2H, t, *J* = 6.6 Hz, C*H*$_2$), 4.10 (2H, t, *J* = 6.6 Hz, C*H*$_2$), 2.26 (2H, quin, *J* = 6.2 Hz, C*H*$_2$), 1.30 (54H, s, stopper C*H*$_3$)

^{13}C NMR (126MHz, $CDCl_3$) δ = 162.9, 162.8, 156.5, 156.2, 148.3, 148.3, 144.1, 144.0, 141.0, 140.3, 139.8, 139.6, 135.9, 133.1, 132.2, 132.1, 131.2, 131.0, 130.7, 130.7, 129.7, 128.4,

128.4, 127.6, 127.5, 127.2, 126.7, 126.7, 126.5, 124.0, 124.0, 113.5, 112.9, 65.9, 63.0, 63.0, 61.7, 53.9, 43.7, 38.6, 34.3, 31.4, 27.9

MS (ESI): m/z calc. for $C_{108}H_{111}N_5O_6I$ $[M+H]^+$: 1701.76; found: 1701.90.

Proto triazolium NDI axle 1·Cl

Proto triazole axle **17** (23 mg, 0.02 mmol, 1 eq.) and Me3O·BF4 (9 mg, 0.06 mmol, 3 eq.) were dissolved in dry CH_2Cl_2 (2 mL). The solution was left to stir for two days at room temperature under N2(g). After this time the reaction was quenched with three drops of MeOH and the solvent was removed *in vacuo*. The resulting solid was purified by silica gel column chromatography (CH_2Cl_2:MeOH 95:5) and the product **1·BF$_4$** isolated as a pale yellow solid. Subsequent anion exchange to yield **1·Cl** was performed by dissolving **1·BF$_4$** in a solvent mixture of 45:45:10 $CHCl_3$:MeOH:H_2O (10 mL) and passing the solution three times through a column of Amberlite® resin loaded with chloride (23 mg, 0.02 mmol, 97%).

^{1}H NMR (500MHz, $CDCl_3$) δ = 9.92 (1H, br. s, triazolium Ar*H*), 8.76 (4H, q, *J* = 8.4 Hz, NDI Ar*H*), 7.67 (2H, br. d, *J* = 6.6 Hz, biphenyl Ar*H*), 7.63 (2H, d, *J* = 8.1 Hz, biphenyl Ar*H*), 7.58 (2H, d, *J* = 7.8 Hz, biphenyl Ar*H*), 7.50 (2H, d, *J* = 7.9 Hz, biphenyl Ar*H*), 7.23 (12H, d, *J* = 8.2 Hz, stopper Ar*H*), 7.14 (2H, d, *J* = 8.5 Hz, stopper Ar*H*), 6.99 - 7.10 (14H, m, stopper Ar*H*), 6.83 (2H, d, *J* = 8.4 Hz, stopper Ar*H*), 6.65 (2H, d, *J* = 8.5 Hz, stopper Ar*H*), 6.01 (2H, br. s., C*H*$_2$), 5.43 (4H, br. s., C*H*$_2$), 4.43 (2H, t, *J* = 6.2 Hz, C*H*$_2$), 4.38 (3H, s, triazolium C*H*$_3$), 4.10 (2H, br. t, *J* = 5.6 Hz, C*H*$_2$), 2.21 - 2.30 (2H, m, C*H*$_2$), 1.24 - 1.37 (54H, m, stopper C*H*$_3$)

^{13}C NMR (126MHz, $CDCl_3$) δ = 162.9, 162.8, 148.5, 148.3, 144.1, 143.7, 142.8, 140.0, 139.6, 136.4, 132.7, 132.2, 131.2, 131.0, 130.7, 130.6, 130.0, 129.8, 128.3, 127.3, 126.8, 126.5, 124.1, 124.0, 113.1, 112.9, 63.1, 63.0, 57.7, 57.6, 38.8, 34.3, 31.9, 31.4, 31.3, 30.9, 30.0, 29.7, 29.4, 27.9, 22.7, 14.1

MS (ESI): m/z calc. for $C_{109}H_{114}N_5O_6$ $[M-Cl]^+$: 1589.8797; found: 1589.8777.

Iodo triazolium NDI axle 2·Cl

Iodo triazole axle **18** (59 mg, 0.03 mmol, 1 eq.) and Me3O·BF4 (16 mg, 0.09 mmol, 3 eq.) were dissolved in dry CH_2Cl_2 (2 mL). The solution was left to stir for two days at room temperature under N2(g). After this time the reaction was quenched with three drops of MeOH and the solvent was subsequently removed *in vacuo*. The resulting solid was purified by silica gel column chromatography (CH_2Cl_2:MeOH 95:5) and the product **2·BF$_4$** isolated as a pale yellow solid. Subsequent anion exchange to yield **2·Cl** was performed by dissolving **2·BF$_4$** in a solvent mixture of 45:45:10 $CHCl_3$:MeOH:H_2O (10 mL) and passing the solution three times through a column of Amberlite® resin loaded with chloride (51 mg, 0.03 mmol, 96%).

^{1}H NMR (400MHz, $CDCl_3$) δ = 8.66 - 8.83 (4H, m, NDI Ar*H*), 7.54 - 7.69 (4H, m, biphenyl Ar*H*), 7.36 - 7.54 (4H, m, biphenyl Ar*H*), 7.23 (12H, d, *J* = 8.1 Hz, stopper Ar*H*), 7.13 - 7.18 (2H, m,

stopper Ar*H*), 6.97 - 7.11 (14H, m, stopper Ar*H*), 6.73 - 6.90 (2H, m, stopper Ar*H*), 6.58 - 6.70 (2H, m, stopper Ar*H*), 5.64 - 5.74 (2H, m, CH_2), 5.41 - 5.50 (2H, m, CH_2), 5.34 - 5.40 (2H, m, CH_2), 4.60 (3H, s, triazolium CH_3), 4.39 - 4.49 (2H, m, CH_2), 4.00 - 4.16 (2H, m, CH_2), 2.19 - 2.31 (2H, m, CH_2), 1.21 - 1.37 (54H, m, stopper CH_3)

^{13}C NMR (126MHz, $CDCl_3$) δ = 162.8, 156.5, 148.5, 148.4, 148.4, 148.3, 144.1, 143.8, 143.8, 143.7, 139.6, 132.7, 132.5, 132.1, 131.2, 131.0, 130.7, 130.6, 130.6, 129.8, 129.7, 129.5, 129.0, 128.2, 127.9, 127.3, 127.3, 127.2, 126.7, 126.7, 124.2, 124.1, 124.0, 113.2, 113.2, 112.9, 63.1, 63.0, 34.3, 31.4, 29.7, 29.6, 27.9, 14.1

MS (ESI): m/z calc. for $C_{109}H_{113}N_5O_6I$ $[M-Cl]^+$: 1715.7764; found: 1715.7697.

Two station HB rotaxane 4·Cl

The two-station axle **1·Cl** (14 mg, 8.6 µmol, 1 eq.) and bis-vinyl nitro macrocycle precursor **3** (8 mg, 12.1 µmol, 1.4 eq.) were dissolved in dry CH_2Cl_2 (2 mL) and stirred for ten minutes. Grubbs' second generation catalyst (1 mg, 10 wt. %) was added and the solution stirred at room temperature, monitoring by TLC and ESI mass spectrometry throughout. After one day a further addition of Grubbs' second generation catalyst (1 mg, 10 wt. %) was made and upon depletion of macrocycle precursor **3** (which occurred after two days) the solvent was removed *in vacuo*. The resulting crude residue was purified by preparative silica TLC (CH_2Cl_2:MeOH 95:5 and EtOAc:MeOH 95:5) to give the product two station rotaxane **1·Cl** as an orange solid (16 mg, 7.1 µmol, 83%).

^{1}H NMR (500MHz, $CDCl_3$) δ = 9.62 (1H, s, internal macrocycle Ar*H*), 9.42 (1H, s, triazolium Ar*H*), 9.14 - 9.32 (2H, m, macrocycle N*H*), 8.76 (2H, s, external macrocycle Ar*H*), 8.77 (4H, q, *J* = 10.5 Hz, NDI Ar*H*), 7.66 (1H, d, *J* = 8.2 Hz, biphenyl Ar*H*), 7.49 (2H, d, *J* = 8.2 Hz, biphenyl Ar*H*), 7.36 (2H, d, *J* = 8.1 Hz, biphenyl Ar*H*), 7.31 (2H, d, *J* = 8.7 Hz, biphenyl Ar*H*), 7.21 - 7.26 (10H, m, stopper Ar*H*), 7.16 (6H, d, *J* = 8.4 Hz, stopper Ar*H*), 6.97 - 7.11 (14H, m, stopper Ar*H*), 6.67 (2H, d, *J* = 8.9 Hz, stopper Ar*H*), 6.30 - 6.45 (8H, m, hydroquinone macrocycle Ar*H*), 5.50 - 5.55 (2H, m, alkene macrocycle C*H*), 5.45 (2H, s, axle CH_2), 5.24 (2H, s, axle CH_2), 4.72 (2H, s, axle CH_2), 4.60 - 4.68 (2H, m, macrocycle CH_2), 4.45 - 4.54 (2H, m, macrocycle CH_2), 4.43 (2H, t, *J* = 6.6 Hz, axle CH_2), 4.10 (2H, t, *J* = 6.6 Hz, axle CH_2), 3.91 - 3.98 (2H, m, macrocycle CH_2), 3.87 (3H, s, triazolium CH_3), 3.62 - 3.86 (12H, m, macrocycle CH_2), 3.49 - 3.58 (2H, m, macrocycle CH_2), 2.21 - 2.30 (2H, m, axle CH_2), 1.31 (54H, d, *J* = 3.5 Hz, stopper CH_3)

^{13}C NMR (126MHz, $CDCl_3$) δ = 164.4, 162.9, 162.9, 156.6, 152.9, 151.9, 148.5, 148.3, 144.1, 144.0, 136.3, 132.5, 132.2, 131.2, 131.1, 130.7, 130.6, 129.9, 129.8, 129.5, 127.5, 127.3, 126.8, 126.7, 126.6, 124.3, 124.0, 114.7, 114.4, 113.5, 113.0, 70.8, 69.4, 67.7, 65.9, 63.2, 63.0, 38.6, 35.6, 35.1, 34.3, 34.3, 31.9, 31.4, 30.1, 29.7, 29.7, 29.4, 28.0, 27.0, 26.9, 26.4, 26.3, 26.2, 22.7, 14.1

MS (ESI): m/z calc. for $C_{141}H_{149}N_8O_{16}$ $[M-Cl]^+$: 2211.1120; found: 2211.1052.

Two station XB rotaxane 5·Cl

The two-station axle **2·Cl** (25 mg, 14.2 µmol, 1 eq.) and bis-vinyl nitro macrocycle precursor **3** (13 mg, 19.9 µmol, 1.4 eq.) were dissolved in dry CH_2Cl_2 (2 mL) and stirred for ten minutes. Grubbs' second generation catalyst (1 mg, 10 wt. %) was added and the solution stirred at room temperature, monitoring by TLC and ESI mass spectrometry throughout. After one day a further addition of Grubbs' second generation catalyst (1 mg, 10 wt. %) was made and after three days the solvent was removed *in vacuo*. The resulting crude residue was purified by repetitive preparative silica TLC (CH_2Cl_2:MeOH 95:5 and EtOAc:MeOH 95:5) to give the product two station rotaxane **5·Cl** as an orange solid (5 mg, 2.1 µmol, 21%).

^{1}H NMR (400MHz, $CDCl_3$) δ = 9.69 (1H, s, internal macrocycle Ar*H*), 9.05 (2H, d, *J* = 1.1 Hz, external macrocycle Ar*H*), 8.83 - 8.92 (2H, m, macrocycle N*H*), 8.76 (4H, q, *J* = 8.1 Hz, NDI Ar*H*), 7.64 - 7.71 (2H, m, biphenyl Ar*H*), 7.54 - 7.63 (2H, m, biphenyl Ar*H*), 7.41 - 7.48 (2H, m, biphenyl Ar*H*), 7.29 - 7.34 (2H, m, biphenyl Ar*H*), 7.14 - 7.25 (14H, m, stopper Ar*H*), 7.00 - 7.10 (14H, m, stopper Ar*H*), 6.95 (2H, dd, *J* = 8.6 Hz, *J* = 5.6 Hz, stopper Ar*H*), 6.67 (2H, d, *J* = 8.9 Hz, stopper Ar*H*), 6.16 - 6.30 (8H, m, hydroquinone macrocycle Ar*H*), 5.50 - 5.54 (2H, m, alkene macrocycle C*H*), 5.44 (2H, s, axle CH_2), 4.88 (2H, s, axle CH_2), 4.63 - 4.77 (2H, m, macrocycle CH_2), 4.41 (2H, s, axle CH_2), 4.37 - 4.46 (4H, m, CH_2 axle CH_2 macrocycle), 4.18 (3H, s, triazolium CH_3), 4.06 - 4.14 (2H, m, axle CH_2), 3.59 - 3.99 (14H, m, macrocycle CH_2), 3.41 - 3.52 (2H, m, macrocycle CH_2), 2.21 - 2.31 (2H, m, axle CH_2), 1.31 (54H, d, *J* = 7.3 Hz, stopper CH_3)

^{13}C NMR (126MHz, $CDCl_3$) δ = 164.4, 162.8, 156.5, 151.9, 148.5, 148.4, 148.4, 148.3, 144.1, 143.8, 143.8, 143.7, 139.6, 136.3, 132.7, 132.5, 132.1, 131.2, 131.0, 130.7, 130.6, 130.6, 129.8, 129.7, 129.5, 129.0, 128.2, 127.9, 127.3, 127.3, 127.2, 126.7, 126.7, 124.2, 124.1, 124.0, 114.7, 114.4, 113.2, 113.2, 112.9, 70.8, 69.4, 67.7, 65.9, 63.1, 63.0, 38.6, 35.6, 34.3, 31.4, 29.7, 29.6, 27.9, 26.4, 26.3, 26.2, 22.7, 14.1

MS (ESI): m/z calc. for $C_{141}H_{148}N_8O_{16}INa$ $[M{-}Cl{+}Na]^{2+}$: 1179.99891; found: 1179.99781

Synthesis of mono station triazolium rotaxanes 6·A and 7·A

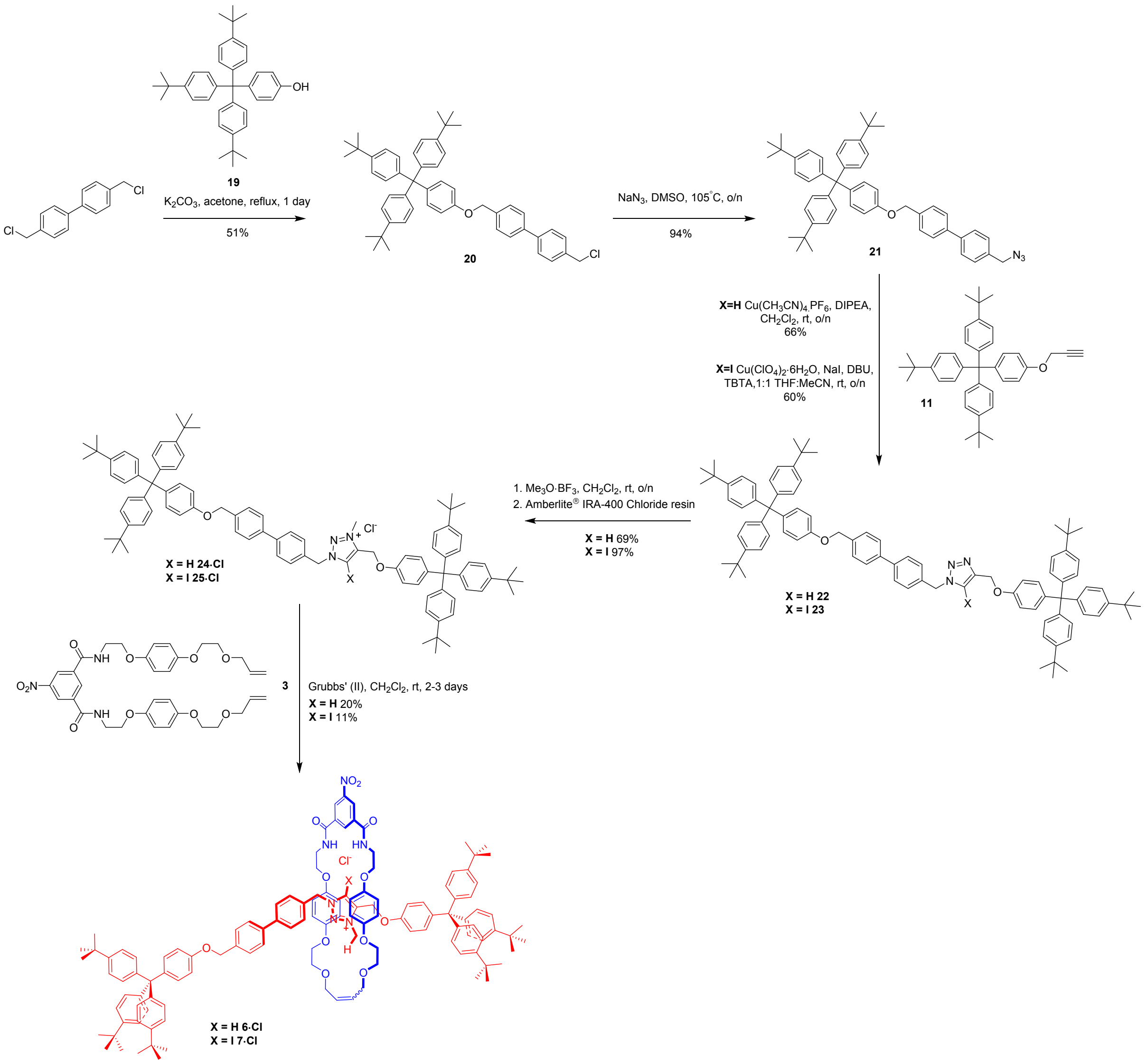

*Scheme S2. Synthetic route to mono-station triazolium rotaxanes **6·Cl** and **7·Cl***

Chloromethyl biphenyl mono stoppered axle pre-cursor 20

Commercially available 4,4'-bis(chloromethyl)-1,1'-biphenyl (186 mg, 0.75 mmol, 1.5 eq.) was dissolved in degassed acetone (30 mL) and to this solution was added dried K_2CO_3 (207 mg, 1.5 mmol, 3 eq.). A solution of alcohol functionalised terphenyl stopper **19** (250 mg, 0.50 mmol, 1 eq.) in acetone (10 mL) was added dropwise before heating the reaction for one day under reflux. After this time the solvent was removed *in vacuo* and the crude solid redissolved in CH_2Cl_2 (50 mL). This was washed with H_2O (3 × 50 mL) and the organic layer isolated and dried over anhydrous $MgSO_4$. Removal of the solvent *in vacuo* was followed by purification by silica gel column chromatography (4:1 petroleum ether:CH_2Cl_2) giving the mono stoppered axle pre cursor **20** (182 mg, 0.25 mmol, 51%).

^{1}H NMR (400MHz, $CDCl_3$) δ = 7.56 - 7.69 (4H, m, biphenyl Ar*H*), 7.43 - 7.56 (4H, m, biphenyl Ar*H*), 7.19 - 7.32 (6H, m, stopper Ar*H*), 7.04 - 7.16 (8H, m, stopper Ar*H*), 6.88 (2H, d, *J* = 7.9 Hz, stopper Ar*H*), 5.09 (2H, s, CH_2), 4.65 (2H, s, CH_2), 1.31 (27H, s, stopper CH_3)

^{13}C NMR (101MHz, $CDCl_3$) δ = 156.6, 148.3, 144.1, 141.0, 140.1, 139.9, 136.5, 136.5, 132.3, 130.7, 129.1, 128.1, 127.5, 127.3, 124.0, 113.3, 69.6, 63.1, 46.0, 34.3, 31.4

MS (ESI): m/z calc. for $C_{51}H_{55}OClK$ $[M+K]^+$: 758.36; found: 758.25

Biphenyl azide mono stoppered axle precursor 21

Compound **20** (114 mg, 0.16 mmol, 1 eq.) and NaN_3 (100 mg, 1.55 mmol, 10 eq.) were suspended in DMSO (40 mL) and heated at 105°C overnight. After cooling H_2O (50 mL) was added to the solution and the resulting precipitate was collected by filtration and dried under vacuum giving the desired axle pre cursor **21** (109 mg, 0.15 mmol, 94%).

^{1}H NMR (400MHz, $CDCl_3$) δ = 7.61 (4H, dd, *J* = 8.3 Hz, *J* = 2.4 Hz, biphenyl Ar*H*), 7.51 (2H, d, *J* = 7.6 Hz, biphenyl Ar*H*), 7.39 (2H, d, *J* = 8.2 Hz, biphenyl Ar*H*), 7.23 (6H, d, *J* = 8.6 Hz, stopper Ar*H*), 7.04 - 7.15 (8H, m, stopper Ar*H*), 6.87 (2H, d, *J* = 8.9 Hz, stopper Ar*H*), 5.08 (2H, s, CH_2), 4.39 (2H, s, CH_2), 1.30 (27H, s, stopper CH_3)

^{13}C NMR (101MHz, $CDCl_3$) δ = 156.6, 148.3, 144.1, 140.8, 140.1, 139.9, 136.5, 134.4, 132.3, 130.7, 128.7, 128.1, 127.5, 127.3, 124.0, 113.3, 69.6, 63.1, 54.5, 34.3, 31.4

MS (ESI): m/z calc. for $C_{51}H_{55}ON_3Na$ $[M+Na]^+$: 749.43; found: 749.34

Proto triazole axle 22

Azide **20** (50 mg, 0.07 mmol, 1 eq.) and the alkyne functionalised terphenyl stopper compound **11** (37 mg, 0.07 mmol, 1 eq.) were dissolved in dry CH_2Cl_2 (3 mL). To this solution were added in order TBTA (7 mg, 0.01 mmol, 0.2 eq.), $Cu(CH_3CN)_4{\cdot}PF_6$ (5 mg, 0.01 mmol, 0.2 eq.) and DIPEA (13 µL, 0.14 mmol, 2 eq.) and the reaction was left to stir overnight at room temperature. After this time the reaction mixture was washed with 25% $NH_4OH_{(aq)}$ (1 × 3 mL) and the organic phase was collected, dried over anhydrous $MgSO_4$ and the solvent removed *in vacuo*. The resulting residue was purified by silica gel column chromatography (CH_2Cl_2) and the proto triazole containing compound **22** was isolated as a white solid (58 mg, 0.05 mmol, 66%).

^{1}H NMR (400MHz, $CDCl_3$) δ = 7.61 (1H, s, triazole Ar*H*), 7.55 - 7.60 (4H, m, biphenyl Ar*H*), 7.51 (2H, d, *J* = 7.7 Hz, biphenyl Ar*H*), 7.36 (2H, d, *J* = 7.6 Hz, biphenyl Ar*H*), 7.20 - 7.26 (12H, m, stopper Ar*H*), 7.02 - 7.15 (16H, m, stopper Ar*H*), 6.86 (4H, t, *J* = 9.1 Hz, stopper Ar*H*), 5.59 (2H, s, CH_2), 5.19 (2H, s, CH_2), 5.08 (2H, s, CH_2), 1.31 (54H, s, stopper CH_3)

^{13}C NMR (101MHz, $CDCl_3$) δ = 156.0, 148.3, 148.3, 144.8, 144.1, 144.0, 139.8, 136.7, 133.5, 132.3, 130.7, 129.0, 128.6, 128.6, 128.0, 127.9, 127.8, 127.2, 124.0, 123.7, 122.6, 113.2, 113.2, 69.5, 63.0, 63.0, 62.0, 54.0, 53.9, 53.4, 34.2, 31.4, 20.5

MS (ESI): m/z calc. for $C_{91}H_{102}N_3O_2$ $[M+H]^+$: 1269.80; found: 1269.89.

Iodo triazole axle 23

Azide **20** (66 mg, 0.07 mmol, 1 eq.) was dissolved in THF (1 mL). NaI (54 mg, 0.28 mmol, 4 eq.) and $Cu(ClO_4)_2 \cdot 6H_2O$ (67 mg, 0.14 mmol, 2 eq.) were added and the resulting suspension was left to stir for five minutes. TBTA (5 mg, 0.01 mmol, 0.1 eq.) and DBU (15 mg, 0.07 mmol, 1 eq.) were added to the mixture. Finally, alkyne functionalised terphenyl stopper compound **11** (49 mg, 0.07 mmol, 1 eq.), and CH_3CN (1 mL) were added and the reaction mixture was left to stir overnight in the dark. The crude mixture was then washed with 25% $NH_4OH_{(aq)}$ (1 × 2 mL) and the organic phase was collected, dried over anhydrous $MgSO_4$ and the solvent removed *in vacuo*. The resulting residue was purified by silica gel column chromatography (CH_2Cl_2). The iodo triazole containing compound **23** was isolated as a white solid (59 mg, 0.04 mmol, 60%).

^{1}H NMR (400MHz, $CDCl_3$) δ = 7.57 (4H, dd, *J* = 8.3 Hz, *J* = 1.6 Hz, biphenyl Ar*H*), 7.49 (2H, d, *J* = 7.8 Hz, biphenyl Ar*H*), 7.37 (2H, d, *J* = 7.7 Hz, biphenyl Ar*H*), 7.19 - 7.26 (12H, m, stopper Ar*H*), 7.04 - 7.15 (16H, m, stopper Ar*H*), 6.82 - 6.93 (4H, m, stopper Ar*H*), 5.65 (2H, s, C*H*$_2$), 5.11 (2H, s, C*H*$_2$), 5.08 (2H, s, C*H*$_2$), 1.30 (54H, s, stopper C*H*$_3$)

^{13}C NMR (101MHz, $CDCl_3$) δ = 148.3, 148.3, 144.3, 144.1, 144.0, 144.0, 134.7, 132.3, 130.7, 130.5, 129.1, 128.6, 128.4, 128.0, 128.0, 127.6, 127.5, 127.3, 124.4, 124.0, 123.7, 113.6, 113.3, 68.4, 63.1, 54.1, 53.4, 47.1, 34.3, 31.4, 30.9, 27.8, 22.2

MS (ESI): m/z calc. for $C_{91}H_{101}N_3O_2I$ $[M+H]^+$: 1395.70; found: 1395.81.

Proto triazolium axle 24·Cl

Proto triazole axle **22** (58 mg, 0.05 mmol, 1 eq.) and $Me_3O \cdot BF_4$ (10 mg, 0.07 mmol, 1.5 eq.) were dissolved in dry CH_2Cl_2 (4 mL). The solution was left to stir for two days at room temperature under $N_{2(g)}$. After this time the reaction was quenched with three drops of MeOH and the solvent was removed *in vacuo*. The resulting solid was purified by silica gel column chromatography (CH_2Cl_2:MeOH 95:5) and the product **24·BF$_4$** isolated as a pale yellow solid. Subseq.uent anion exchange to yield **24·Cl** was performed by dissolving **24·BF$_4$** in a solvent mixture of 45:45:10 $CHCl_3$:MeOH:H_2O (10 mL) and passing the solution three times through a column of Amberlite® resin loaded with chloride (46 mg, 0.04 mmol, 69%).

^{1}H NMR (400MHz, $CDCl_3$) δ = 8.46 (1H, s, triazolium Ar*H*), 7.62 - 7.66 (2H, m, biphenyl Ar*H*), 7.47 - 7.58 (6H, m, biphenyl Ar*H*), 7.19 - 7.26 (12H, m, stopper Ar*H*), 7.01 - 7.17 (16H, m, stopper Ar*H*), 6.77 - 6.89 (4H, m, stopper Ar*H*), 5.74 (2H, s, C*H*$_2$), 5.27 (2H, s, C*H*$_2$), 5.07 (2H, s, C*H*$_2$), 4.32 (3H, s, triazolium C*H*$_3$), 1.30 (54H, s, stopper C*H*$_3$)

^{13}C NMR (126MHz, $CDCl_3$) δ = 156.6, 148.4, 148.3, 148.2, 144.3, 144.1, 143.7, 140.0, 139.5, 139.4, 136.9, 132.7, 132.3, 130.7, 130.6, 130.1, 129.7, 129.2, 128.1, 128.0, 127.4, 127.3, 126.6, 124.1, 124.0, 124.0, 113.2, 113.2, 72.3, 69.5, 63.1, 63.0, 38.9, 34.3, 34.2, 31.3

MS (MALDI-TOF): *m/z* calc. for $C_{92}H_{104}N_3O_2$ $[M - Cl]^+$: 1284.82; found: 1284.69.

Iodo triazolium axle 25·Cl

Iodo triazole axle **23** (59 mg, 0.04 mmol, 1 eq.) and $Me_3O \cdot BF_4$ (10 mg, 0.07 mmol, 1.5 eq.) were dissolved in dry CH_2Cl_2 (30 mL). The solution was left to stir for two days at room temperature under $N_{2(g)}$. After this time the reaction was quenched with three drops of MeOH and the solvent was removed *in vacuo*. The resulting solid was purified by silica gel column chromatography (CH_2Cl_2:MeOH 95:5) and the product **25·BF$_4$** isolated as a white solid. Subsequent anion exchange to yield **25·Cl** was performed by dissolving **25·BF$_4$** in a solvent mixture of 45:45:10 $CHCl_3$:MeOH:H_2O (10 mL) and passing the solution three times through a column of Amberlite® resin loaded with chloride (56 mg, 0.04 mmol, 97%).

^{1}H NMR (400MHz, $CDCl_3$) δ = 7.46 - 7.56 (2H, m, biphenyl Ar*H*), 7.31 - 7.46 (6H, m, biphenyl Ar*H*), 7.15 - 7.25 (12H, m, stopper Ar*H*), 6.95 - 7.15 (16H, m, stopper Ar*H*), 6.65 - 6.86 (4H, m, stopper Ar*H*), 5.70 (2H, s, C*H*$_2$), 5.36 (2H, s, C*H*$_2$), 4.99 (2H, s, C*H*$_2$), 4.42 (3H, s, triazolium C*H*$_3$), 1.27 (54H, s, stopper C*H*$_3$)

^{13}C NMR (126MHz, $CDCl_3$) δ = 156.5, 154.5, 148.4, 148.3, 144.1, 143.8, 143.3, 142.1, 141.8, 139.9, 139.2, 136.8, 132.6, 132.3, 130.7, 130.6, 128.9, 128.0, 128.0, 127.2, 124.2, 124.1, 124.0, 113.3, 113.2, 104.6, 69.4, 63.1, 63.0, 59.5, 57.1, 39.7, 34.3, 34.3, 31.4, 29.7

MS (MALDI-TOF): *m/z* calc. for $C_{92}H_{103}IN_3O_2$ $[M - Cl]^+$: 1410.72; found: 1410.97.

HB one station rotaxane 6·Cl

The one-station axle **24·Cl** (30 mg, 22.7 µmol, 1 eq.) and bis-vinyl nitro macrocycle precursor **3** (18 mg, 27.2 µmol, 1.2 eq.) were dissolved in dry CH_2Cl_2 (2 mL) and stirred for ten minutes. Grubbs' second generation catalyst (2 mg, 10 wt. %) was added and the solution stirred at room temperature, monitoring by TLC and ESI mass spectrometry throughout. After one day a further addition of Grubbs' second generation catalyst (2 mg, 10 wt. %) was made and upon depletion of macrocycle precursor **3** (which occurred after two days) the solvent was removed *in vacuo*. The resulting crude residue was purified by preparative silica TLC (EtOAc:MeOH 99:1 and CH_2Cl_2:MeOH 98:2) to give the product one station rotaxane **6·Cl** as an off white solid (9 mg, 4.6 µmol, 20%).

^{1}H NMR (500MHz, $CDCl_3$) δ = 9.69 (1H, br. s, internal macrocycle Ar*H*), 9.49 (1H, br. s, triazolium Ar*H*), 9.26 - 9.42 (2H, m, macrocycle N*H*), 8.80 (2H, s, external macrocycle Ar*H*), 7.55 (4H, d, *J* = 4.1 Hz, biphenyl Ar*H*), 7.42 (2H, d, *J* = 8.1 Hz, biphenyl Ar*H*), 7.32 (2H, d, *J* = 8.7 Hz, biphenyl Ar*H*), 7.20 - 7.27 (12H, m, stopper Ar*H*), 7.06 - 7.20 (16H, m, stopper Ar*H*), 7.01 (2H, d, *J* = 8.7 Hz, stopper Ar*H*), 6.88 (2H, d, *J* = 8.9 Hz, stopper Ar*H*), 6.32 - 6.49 (8H, m, hydroquinone macrocycle Ar*H*), 5.54 (2H, br. s., alkene macrocycle C*H*), 5.27 (2H, s, axle C*H*$_2$), 5.09 (2H, s, axle C*H*$_2$), 4.72 (2H, s, axle C*H*$_2$), 4.56 - 4.68 (2H, m, macrocycle C*H*$_2$), 4.45 - 4.56 (2H, m, macrocycle C*H*$_2$), 3.94 - 4.00 (2H, m, macrocycle C*H*$_2$), 3.88 (3H, s, triazolium

CH_3), 3.63 - 3.92 (12H, m, macrocycle CH_2), 3.52 - 3.62 (2H, m, macrocycle CH_2), 1.31 (54H, s, stopper CH_3)

^{13}C NMR (126MHz, $CDCl_3$) δ = 164.1, 153.0, 151.9, 148.5, 148.3, 144.1, 144.0, 132.5, 132.3, 130.9, 130.7, 130.6, 129.8, 129.6, 128.8, 128.2, 127.5, 127.2, 126.6, 124.3, 124.0, 115.0, 114.4, 113.8, 113.3, 70.8, 69.4, 68.1, 67.6, 66.5, 63.1, 39.9, 38.9, 38.7, 34.3, 34.3, 31.9, 31.4, 30.3, 29.7, 29.7, 29.4, 28.9, 23.7, 23.0, 22.7, 14.1, 14.0, 10.9

MS (ESI): m/z calc. for $C_{124}H_{139}N_6O_{12}$ [M−Cl]$^+$: 1905.0524; found: 1905.0453.

XB one station rotaxane 7·Cl

The one-station axle **25·Cl** (33 mg, 22.8 µmol, 1 eq.) and bis-vinyl nitro macrocycle precursor **3** (18 mg, 27.4 µmol, 1.4 eq.) were dissolved in dry CH_2Cl_2 (2 mL) and stirred for ten minutes. Grubbs' second generation catalyst (2 mg, 10 wt. %) was added and the solution stirred at room temperature, monitoring by TLC and ESI mass spectrometry throughout. After one day a further addition of Grubbs' second generation catalyst (2 mg, 10 wt. %) was made and after three days the solvent was removed *in vacuo*. The resulting crude residue was purified by iterative preparative silica TLC (CH_2Cl_2:MeOH 97:3) to give the product one station rotaxane **7·Cl** as a white solid (5 mg, 2.4 µmol, 11%).

^{1}H NMR (400MHz, $CDCl_3$) δ = 9.35 (1H, br. s, internal macrocycle Ar*H*), 9.01 (2H, s, external macrocycle Ar*H*), 8.38 - 8.54 (2H, m, macrocycle N*H*), 7.63 - 7.68 (2H, m, biphenyl Ar*H*), 7.53 - 7.58 (2H, m, biphenyl Ar*H*), 7.48 - 7.52 (2H, m, biphenyl Ar*H*), 7.30 - 7.35 (2H, m, biphenyl Ar*H*), 7.15 - 7.26 (12H, m, stopper Ar*H*), 7.06 - 7.14 (16H, m, stopper Ar*H*), 6.97 (2H, d, *J* = 2.4 Hz, stopper Ar*H*), 6.88 (2H, d, *J* = 8.9 Hz, stopper Ar*H*), 6.29 - 6.39 (8H, m, hydroquinone macrocycle Ar*H*), 5.40 - 5.51 (2H, m, alkene macrocycle C*H*), 5.09 (2H, s, axle CH_2), 4.88 (2H, s, axle CH_2), 4.67 - 4.80 (2H, m, macrocycle CH_2), 4.51 - 4.60 (2H, m, macrocycle CH_2), 4.49 (2H, s, axle CH_2), 4.15 (3H, s, triazolium CH_3), 3.97 - 4.06 (2H, m, macrocycle CH_2), 3.85 - 3.95 (2H, m, macrocycle CH_2), 3.59 - 3.83 (10H, m, macrocycle CH_2), 3.45 - 3.56 (2H, m, macrocycle CH_2), 1.32 (54H, s, stopper CH_3)

^{13}C NMR (126MHz, $CDCl_3$) δ = 164.4, 155.1, 153.0, 151.6, 148.5, 148.3, 144.1, 143.9, 142.8, 139.9, 135.9, 132.5, 132.3, 130.7, 130.7, 130.6, 130.2, 129.2, 128.2, 127.9, 127.4, 126.7, 124.3, 124.0, 115.8, 115.4, 114.7, 114.1, 113.2, 70.7, 69.6, 69.4, 67.6, 66.9, 63.2, 63.1, 39.8, 39.5, 37.1, 34.3, 34.3, 32.7, 31.9, 31.4, 30.0, 29.7, 29.7, 29.4, 27.1, 22.7, 19.7, 14.1

MS (ESI): *m/z* calc. for $C_{124}H_{138}IN_6O_{12}$ [M − Cl]$^+$: 2031.9524; found: 2031.9476.

Synthesis of mono station NDI rotaxane 8

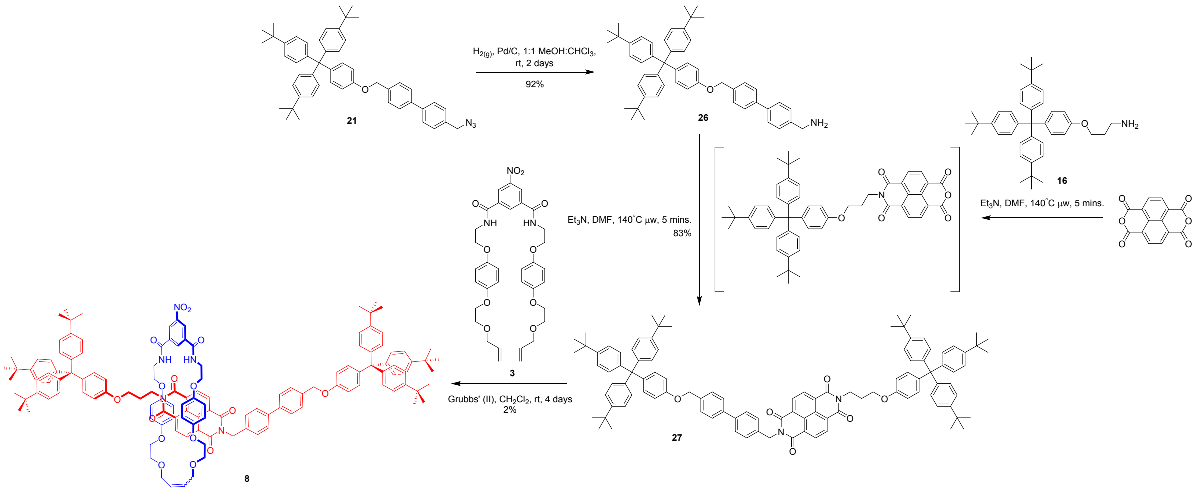

*Scheme S3. Synthetic route to mono-station NDI rotaxane **8***

Biphenyl amine mono stoppered axle pre-cursor 26

Azide **21** (200 mg, 0.28 mmol) was dissolved in $CHCl_3$ (10 mL) and Palladium on Carbon (40 mg, 20 wt. %) was added to the solution followed by MeOH (10 mL). The reaction was stirred under an atmosphere of $H_{2(g)}$ for two days after which the crude mixture was filtered through a plug of Celite, which was rinsed with a further 50 mL of 1:1 $CHCl_3$:MeOH to give a clear solution. The solvent was then removed *in vacuo* and the free amine **26** isolated by silica gel column chromatography (CH_2Cl_2:MeOH 95:5) as a white solid (180 mg, 0.26 mmol, 92%).

^{1}H NMR (500MHz, MeOD) δ = 7.55 - 7.62 (4H, m, biphenyl Ar*H*), 7.44 - 7.51 (2H, m, biphenyl Ar*H*), 7.34 - 7.42 (2H, m, biphenyl Ar*H*), 7.17 - 7.26 (6H, m, stopper Ar*H*), 7.01 - 7.12 (8H, m, stopper Ar*H*), 6.82 - 6.89 (2H, m, stopper Ar*H*), 5.07 (2H, s, C*H*$_2$), 4.17 (2H, br. s, N*H*$_2$), 3.86 (2H, s, C*H*$_2$), 1.27 (27H, s, stopper C*H*$_3$)

^{13}C NMR (126MHz, MeOD) δ = 156.3, 148.1, 143.9, 140.1, 139.7, 136.1, 132.0, 130.5, 127.8, 127.7, 127.1, 126.9, 123.8, 113.1, 69.5, 62.8, 44.8, 34.0, 31.0

MS (ESI): *m/z* calc. for $C_{51}H_{58}NO$ $[M + H]^+$: 700.45; found: 700.41.

NDI mono station axle 27

The propylamine functionalised terphenyl stopper compound **16** (40 mg, 0.07 mmol, 1 eq.) and commerically available 1,4,5,8-naphthalenetetracarboxylic dianhydride (19 mg, 0.07 mmol, 1 eq.) were suspended in 1 mL of dry, degassed DMF with dry Et_3N (6 µL, 0.07 mmol, 1 eq.) in a sealed microwave vial. The suspension was sonicated for ten minutes before heating under microwave irradiation for five minutes at 140°C. The resulting brown solution was allowed to cool and the vial opened to allow for the addition of amine **26** (50 mg, 0.07

mmol, 1 eq.) and further dry Et_3N (6 µL, 0.07 mmol, 1 eq.). The vial was resealed and the suspension sonicated for 30 minutes before heating under microwave irradiation for a further five minutes at 140°C. Upon cooling the brown solution was placed immediately into a round bottom flask and reduced *in vacuo* to give a golden brown residue. This crude reaction mixture was purified by silica gel column chromatography (CH_2Cl_2) to yield the desired one station axle compound **27** (89 mg, 0.06 mmol, 83%).

^{1}H NMR (400MHz, $CDCl_3$) δ = 8.71 - 8.83 (4H, m, NDI Ar*H*), 7.63 (2H, s, biphenyl Ar*H*), 7.61 - 7.68 (2H, m, biphenyl Ar*H*), 7.52 - 7.59 (4H, m, biphenyl Ar*H*), 7.45 - 7.50 (2H, m, biphenyl Ar*H*), 7.19 - 7.26 (12H, m, stopper Ar*H*), 6.96 - 7.14 (16H, m, stopper Ar*H*), 6.80 - 6.91 (2H, m, stopper Ar*H*), 6.57 - 6.69 (2H, m, stopper Ar*H*), 5.45 (2H, s, CH_2), 5.06 (2H, s, CH_2), 4.44 (2H, t, *J* = 7.0 Hz, CH_2), 4.11 (2H, t, *J* = 6.1 Hz, CH_2), 2.21 - 2.33 (2H, m, CH_2), 1.30 (54H, s, stopper CH_3)

^{13}C NMR (126MHz, $CDCl_3$) δ = 162.9, 162.8, 162.8, 156.6, 156.5, 148.3, 144.1, 140.4, 140.3, 139.9, 139.6, 136.3, 135.6, 132.3, 132.1, 131.2, 131.0, 130.7, 130.7, 129.7, 129.6, 128.0, 127.3, 127.2, 126.7, 126.7, 126.6, 124.0, 113.2, 112.9, 69.6, 65.9, 63.0, 63.0, 43.7, 38.6, 34.3, 31.4, 27.9

MS (MALDI-TOF): *m/z* calc. for $C_{105}H_{109}N_2O_6$ $[M + H]^+$: 1495.83; found: 1495.66.

NDI mono station rotaxane 8

The one-station axle **27** (45 mg, 30.1 µmol, 1 eq.) and bis-vinyl nitro macrocycle precursor **3** (30 mg, 45.2 µmol, 1.5 eq.) were dissolved in dry CH_2Cl_2 (2 mL) and stirred for ten minutes. Grubbs' second generation catalyst (3 mg, 10 wt. %) was added and the solution stirred at room temperature, monitoring by TLC throughout. After one day a further addition of Grubbs' second generation catalyst (3 mg, 10 wt. %) was made and upon depletion of macrocycle precursor **3** (which occurred after four days) the solvent was removed *in vacuo*. The resulting crude residue was purified by preparative silica TLC (CH_2Cl_2:MeOH 99:1) to give the product one station rotaxane **8** as an orange solid (1 mg, 0.47 µmol, 2%).

^{1}H NMR (500MHz, $CDCl_3$) δ = 9.19 (1H, s, internal macrocycle Ar*H*), 9.10 (2H, s, external macrocycle Ar*H*), 8.50 - 8.61 (4H, m, NDI Ar*H*), 7.53 - 7.63 (6H, m, biphenyl Ar*H*), 7.46 - 7.51 (2H, m, biphenyl Ar*H*), 7.18 - 7.25 (12H, m, stopper Ar*H*), 7.03 - 7.13 (16H, m, stopper Ar*H*), 6.83 - 6.89 (2H, m, stopper Ar*H*), 6.71 - 6.77 (2H, m, stopper Ar*H*), 6.24 - 6.33 (2H, m, alkene macrocycle C*H*), 5.63 - 5.80 (8H, m, hydroquinone macrocycle Ar*H*), 5.27 (2H, s, axle CH_2), 5.05 (2H, s, CH_2 axle), 4.31 (2H, t, *J* = 7.0 Hz, CH_2 axle), 4.17 - 4.26 (4H, m, macrocycle CH_2), 3.98 - 4.17 (6H, m, macrocycle and axle CH_2), 3.37 - 3.81 (12H, m, macrocycle CH_2), 2.20 - 2.29 (2H, m, axle CH_2), 1.30 (54H, s, stopper CH_3)

^{13}C NMR (126MHz, $CDCl_3$) δ = 164.9, 163.5, 163.1, 156.6, 156.5, 152.4, 151.3, 149.2, 148.3, 148.3, 144.1, 144.0, 140.8, 140.1, 139.9, 136.6, 136.2, 135.3, 132.3, 131.1, 131.0, 130.7, 130.6, 130.2, 130.0, 129.7, 128.6, 128.2, 128.2, 127.2, 126.7, 126.0, 125.9, 125.6, 124.0,

114.1, 114.0, 113.9, 113.2, 113.1, 71.1, 69.6, 68.8, 67.2, 66.9, 65.9, 63.0, 63.0, 44.0, 40.7, 38.5, 34.3, 31.4, 29.7, 28.1

MS (MALDI-TOF): *m/z* calc. for $C_{137}H_{143}N_5O_{16}Na$ $[M + Na]^+$: 2139.05; found: 2139.54

Part II: 2D ^{1}H NMR ROESY spectra

2D ^{1}H NMR ROESY of 4·Cl

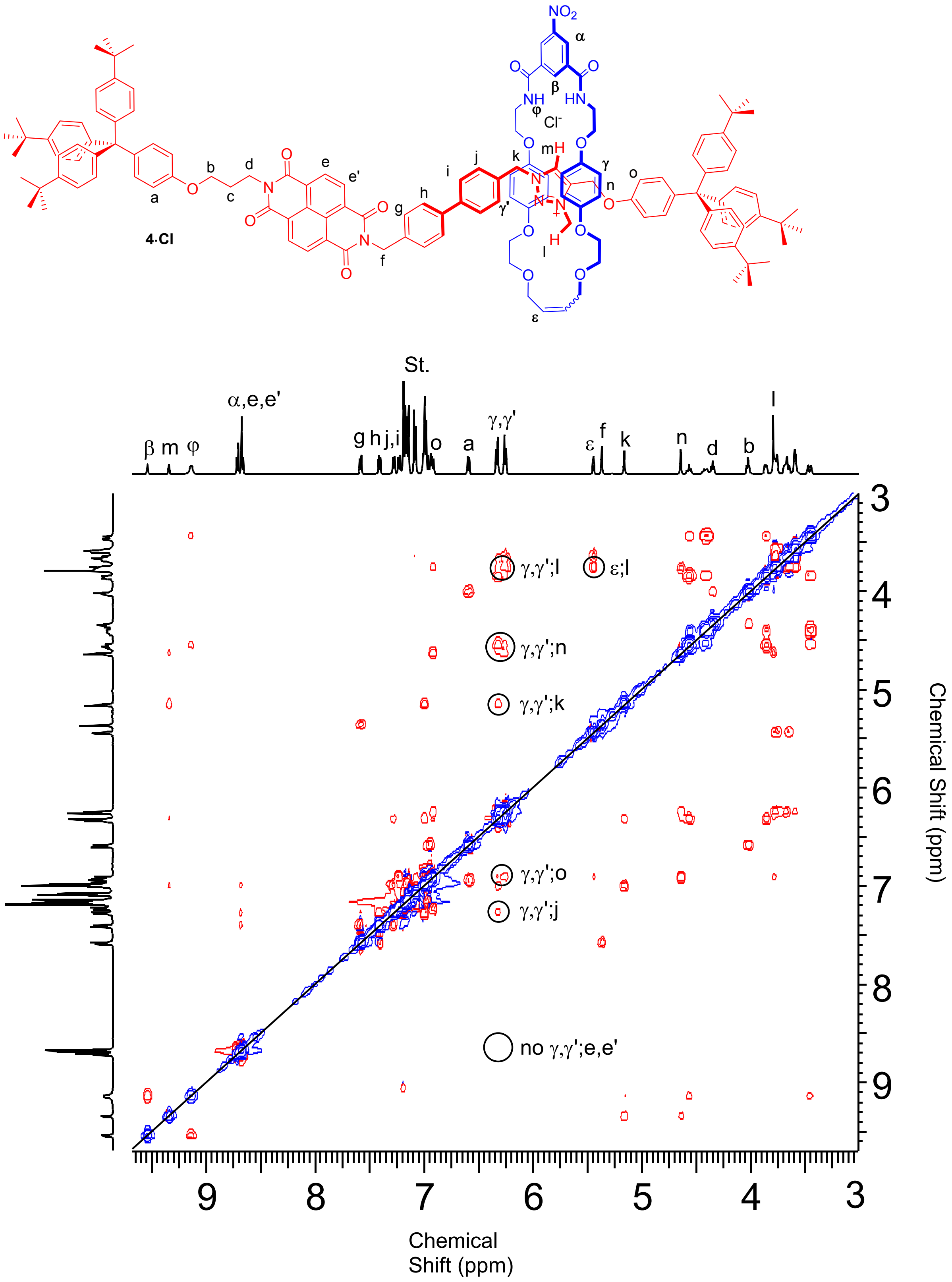

*Figure S1. Truncated ^{1}H-^{1}H ROESY NMR spectrum of rotaxane **4·Cl** with selected coupling interactions highlighted ($CDCl_3$, 298 K, 500 MHz).*

2D ^{1}H NMR ROESY of 4·PF_6

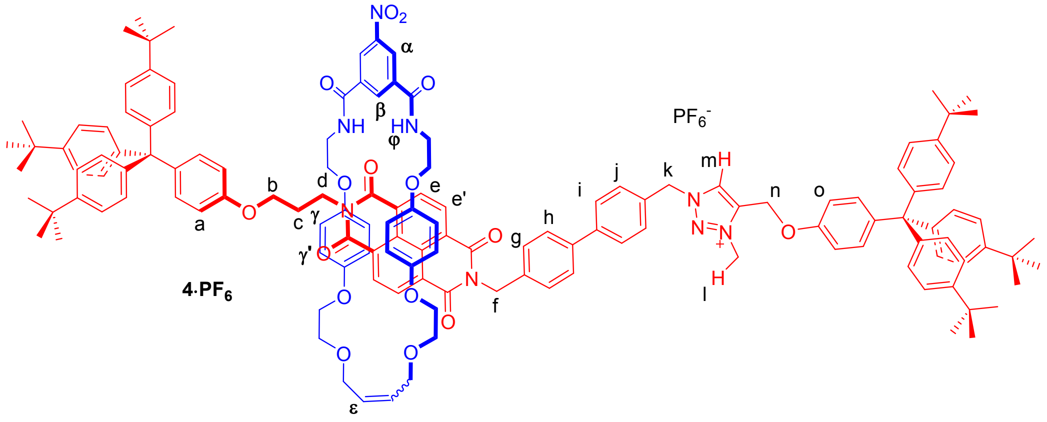

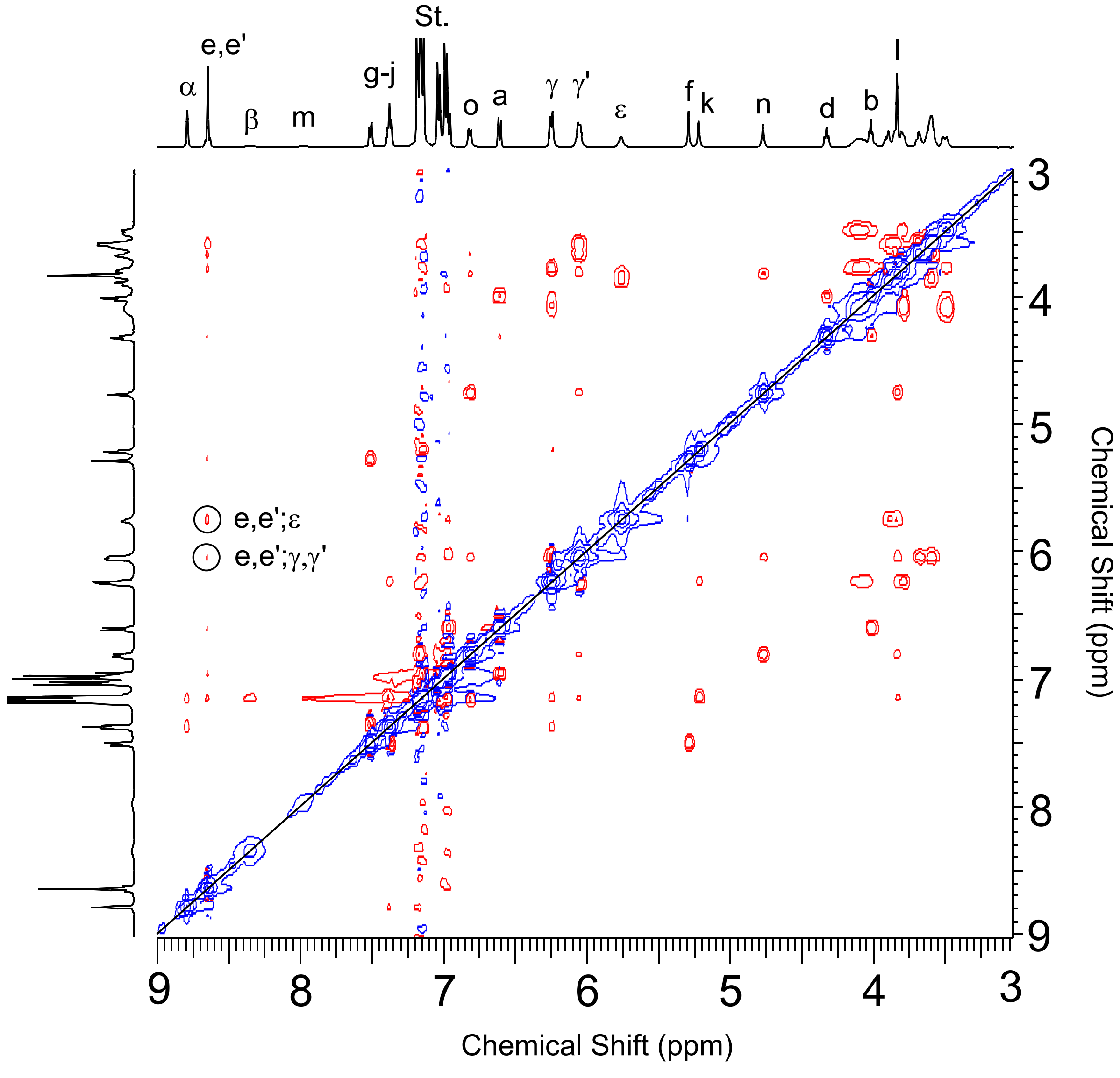

*Figure S2. Truncated ^{1}H-^{1}H ROESY NMR spectrum of rotaxane **4·PF_6** with selected coupling interactions highlighted ($CDCl_3$, 298 K, 500 MHz).*

2D ^{1}H NMR ROESY of 5·Cl

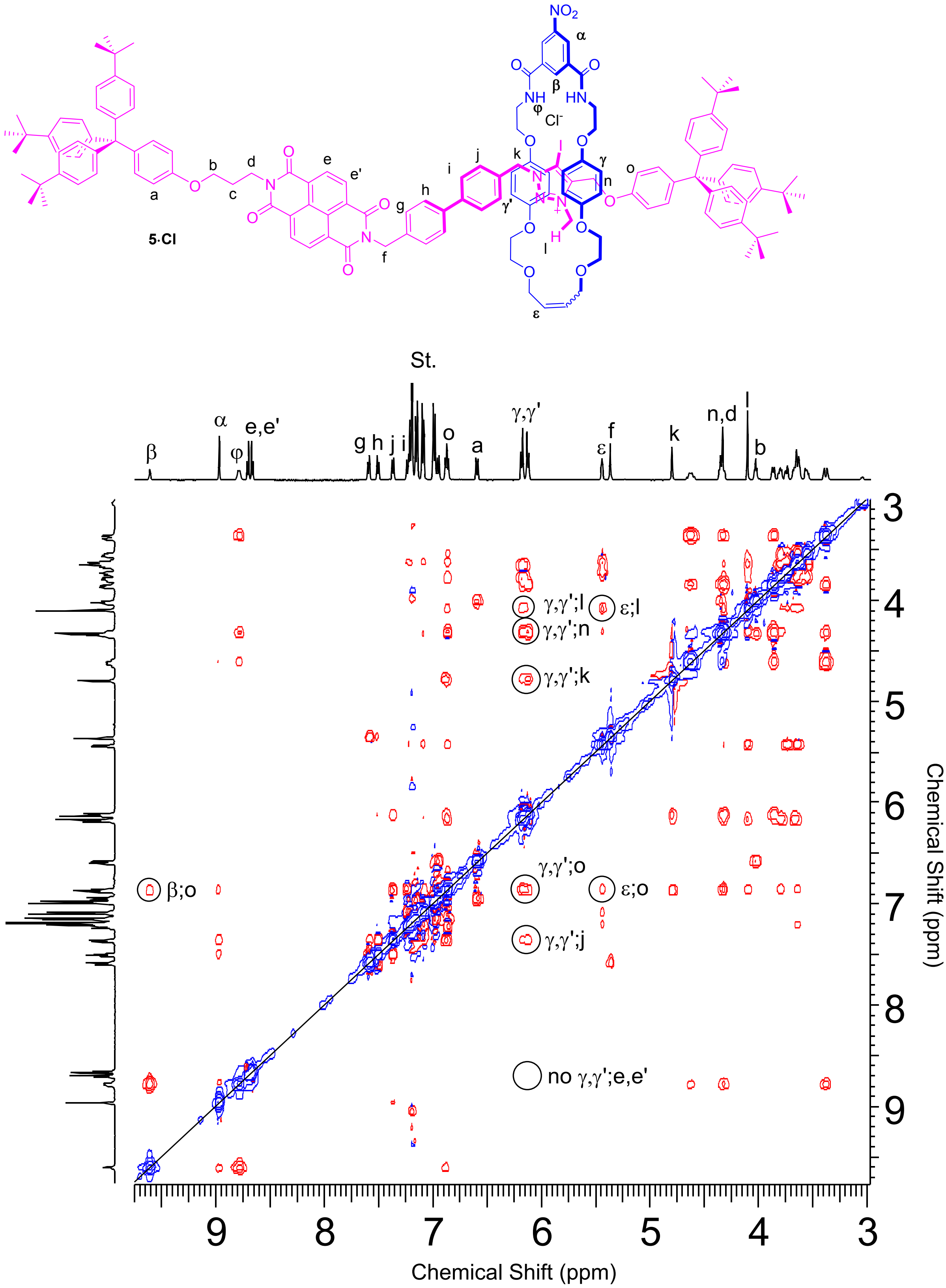

*Figure S3. Truncated ^{1}H-^{1}H ROESY NMR spectrum of rotaxane **5·Cl** with selected coupling interactions highlighted ($CDCl_3$, 298 K, 500 MHz).*

2D ^{1}H NMR ROESY of 5·PF_6

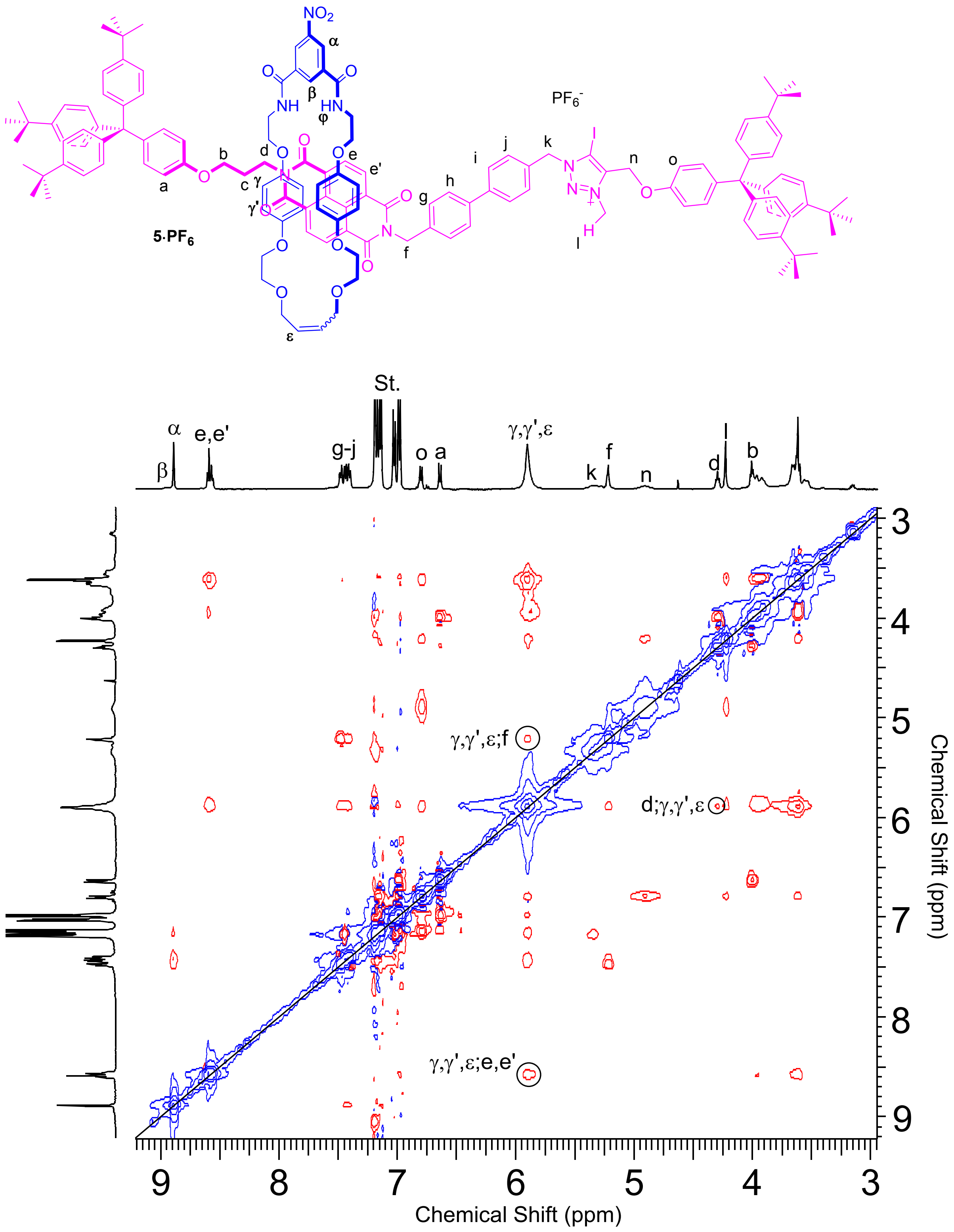

*Figure S4. Truncated ¹H-¹H ROESY NMR spectrum of rotaxane **5·PF$_6$** with selected coupling interactions highlighted (CDCl$_3$, 298 K, 500 MHz).*

Part III: UV-Vis Spectroscopy

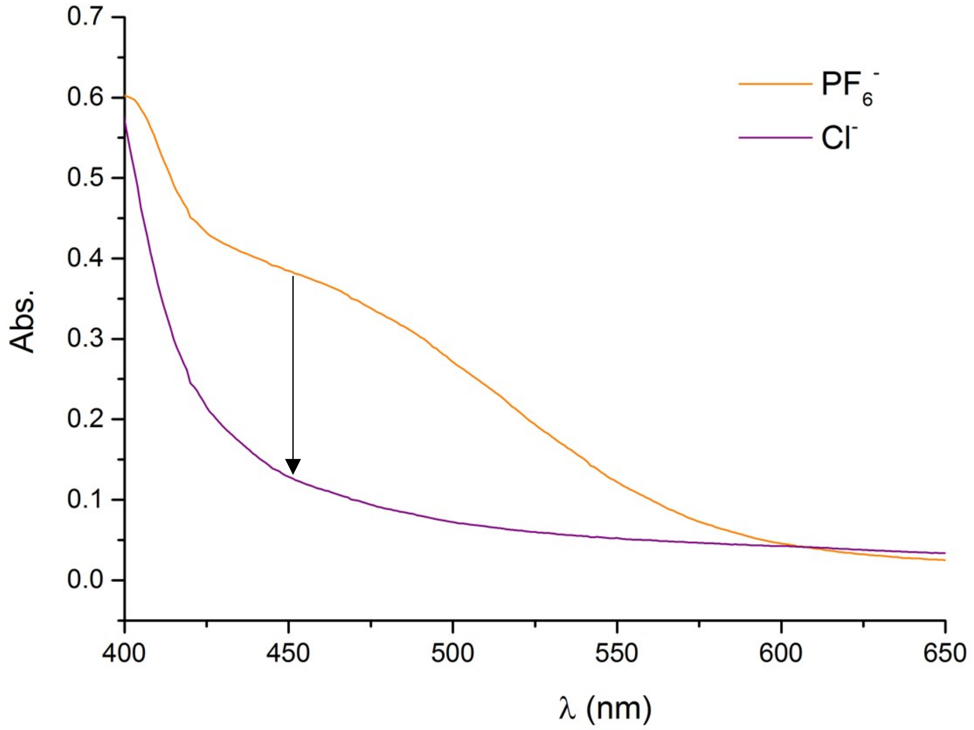

Figure S5: UV-Vis spectra showing the effect of the addition of one equivalent of tetrabutylammonium chloride into a solution of rotaxane ***5·PF₆*** *in $CHCl_3$ (3.3×10^{-3} mol L^{-1}). The addition of chloride leads to a decrease in intensity of the charge-transfer band at $\lambda_{abs,max}$ ~ 450 nm.*

Part IV: Anion molecular motion studies

Axle and rotaxane components were fully anion exchanged using a column containing an Amberlite® ion exchange resin as described in the general information and characterised by a combination of ^{1}H, ^{19}F and ^{31}P NMR spectroscopy.

Two station HB axle 1·A

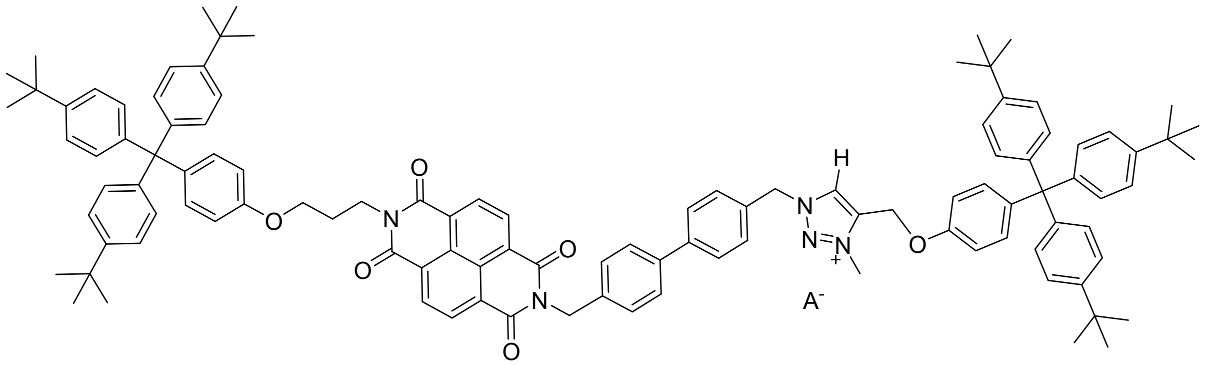

1·I

^{1}H NMR (400MHz, 4:1 $CDCl_3$:MeOD) δ = 8.88 (1H, s, triazolium Ar*H*), 8.63 - 8.79 (4H, m, NDI Ar*H*), 7.44 - 7.62 (8H, m, biphenyl Ar*H*), 6.91 - 7.24 (28H, m, stopper Ar*H*), 6.81 (2H, d, *J* = 9.0 Hz, stopper Ar*H*), 6.56 (2H, d, *J* = 8.9 Hz, stopper Ar*H*), 5.83 (2H, s, CH_2), 5.39 (2H, s, CH_2), 5.33 (2H, s, CH_2), 4.35 (2H, s, CH_2), 4.35 (3H, s, triazolium CH_3), 4.07 (2H, s, CH_2), 2.16 - 2.28 (2H, m, CH_2), 1.23 (54H, s, stopper CH_3)

1·PF_6

^{1}H NMR (500MHz, $CDCl_3$) δ = 8.69 - 8.84 (4H, m, NDI Ar*H*), 8.35 (1H, s, triazolium Ar*H*), 7.45 - 7.68 (8H, m, biphenyl Ar*H*), 6.98 - 7.26 (28H, m, stopper Ar*H*), 6.78 (2H, d, *J* = 8.9 Hz, stopper Ar*H*), 6.65 (2H, d, *J* = 8.7 Hz, stopper Ar*H*), 5.69 (2H, s, CH_2), 5.42 (2H, s, CH_2), 5.23 (2H, s, CH_2), 4.38 - 4.48 (2H, m, CH_2), 4.33 (3H, s, triazolium CH_3), 4.10 (2H, s, CH_2), 2.20 - 2.28 (2H, m, CH_2), 1.30 (54H, s, stopper CH_3)

^{19}F NMR (470 MHz, $CDCl_3$) δ = -72.5 (d, ^{1}J = 708 Hz, PF_6).

^{31}P NMR (202 MHz, $CDCl_3$) δ = -144.3 (sept, ^{1}J = 709 Hz, PF_6).

Two station XB axle 2·A

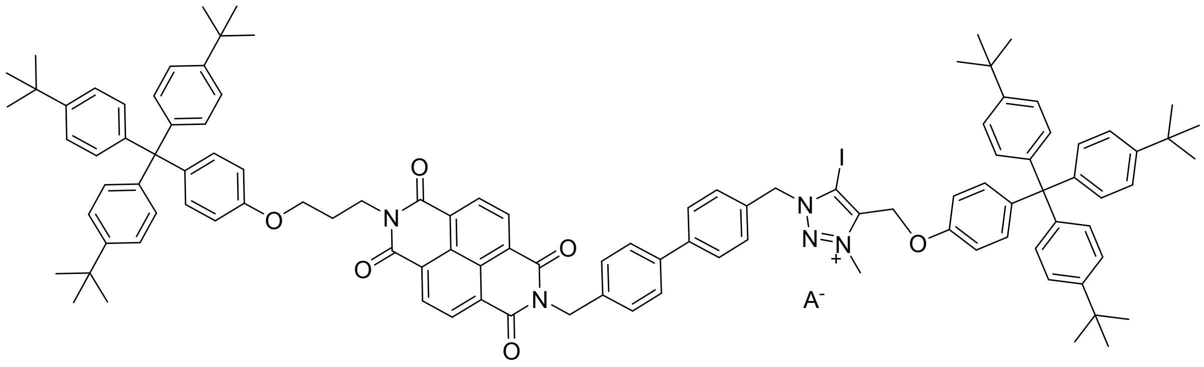

2·I

^{1}H NMR (400MHz, 4:1 $CDCl_3$:MeOD) δ = 8.63 - 8.77 (4H, m, NDI Ar*H*), 7.42 - 7.60 (8H, m, biphenyl Ar*H*), 6.91 - 7.25 (28H, m, stopper Ar*H*), 6.79 - 6.86 (2H, m, stopper Ar*H*), 6.51 - 6.59 (2H, m, stopper Ar*H*), 5.83 (2H, s, CH_2), 5.38 (2H, s, CH_2), 5.28 (2H, s, CH_2), 4.38 (3H, s, triazolium CH_3), 4.33 - 4.42 (2H, m, CH_2), 4.03 - 4.11 (2H, m, CH_2), 2.17 - 2.28 (2H, m, CH_2), 1.25 (54H, s, stopper CH_3)

2·PF$_6$

^{1}H NMR (500MHz, $CDCl_3$) δ = 8.65 - 8.83 (4H, m, NDI Ar*H*), 7.38 - 7.66 (8H, m, biphenyl Ar*H*), 6.98 - 7.26 (28H, m, stopper Ar*H*), 6.74 - 6.88 (2H, m, stopper Ar*H*), 6.60 - 6.69 (2H, m, stopper Ar*H*), 5.74 (2H, s, CH_2), 5.44 (2H, s, CH_2), 5.30 (2H, s, CH_2), 4.38 - 4.48 (2H, m, CH_2), 4.34 (3H, s, triazolium CH_3), 4.10 (2H, br. s., CH_2), 2.20 - 2.31 (2H, m, CH_2), 1.30 (54H, s, stopper CH_3)

^{19}F NMR (470 MHz, $CDCl_3$) δ = -72.5 (d, ^1J = 708 Hz, PF_6).

^{31}P NMR (202 MHz, $CDCl_3$) δ = -144.3 (sept, ^1J = 709 Hz, PF_6).

Two station HB rotaxane 4·A

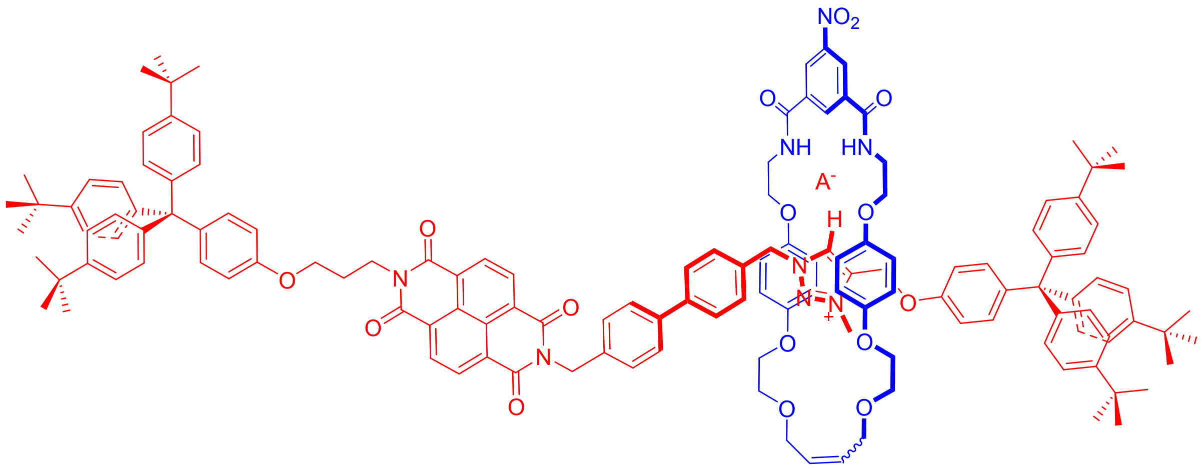

4·I

^{1}**H NMR** (400MHz, 4:1 $CDCl_3$:MeOD) δ = 9.19 (1H, s, internal macrocycle Ar*H*), 8.74 - 8.82 (3H, m, triazolium and external macrocycle Ar*H*), 8.62 - 8.71 (4H, m, NDI Ar*H*), 8.44 - 8.49 (2H, m, macrocycle N*H*), 7.39 - 7.59 (6H, m, biphenyl Ar*H*), 7.23 - 7.28 (2H, m, biphenyl Ar*H*), 6.96 - 7.23 (28H, m, stopper Ar*H*), 6.88 - 6.93 (2H, m, stopper Ar*H*), 6.59 - 6.65 (2H, m, stopper Ar*H*), 6.03 - 6.29 (8H, m, hydroquinone macrocycle Ar*H*), 5.78 - 5.87 (2H, m, alkene macrocycle C*H*), 5.45 (2H, s, axle CH_2), 5.31 (2H, s, axle CH_2), 4.96 (2H, s, axle CH_2), 4.30 - 4.42 (4H, m, axle and macrocycle CH_2), 4.00 (3H, s, triazolium CH_3), 3.81 - 4.15 (14H, m, axle and macrocycle CH_2), 3.56 - 3.77 (8H, m, macrocycle CH_2), 2.13 - 2.28 (2H, m, axle CH_2), 1.24 (54H, s, stopper CH_3)

4·PF$_6$

^{1}**H NMR** (500MHz, $CDCl_3$) δ = 8.87 (2H, s, external macrocycle Ar*H*), 8.67 - 8.78 (4H, m, NDI Ar*H*), 8.43 (1H, br. s, internal macrocycle Ar*H*), 8.07 (1H, br. s, triazolium), 7.54 - 7.64 (2H, m, biphenyl Ar*H*), 7.40 - 7.52 (4H, m, biphenyl Ar*H*), 6.97 - 7.32 (30H, m, stopper and biphenyl Ar*H*), 6.85 - 6.94 (2H, m, stopper Ar*H*), 6.69 (2H, d, *J* = 8.9 Hz, stopper Ar*H*), 6.33 (8H, d, *J* = 8.4 Hz, hydroquinone macrocycle Ar*H*), 5.77 - 5.92 (2H, m, alkene macrocycle C*H*), 5.37 (2H, s, axle CH_2), 5.30 (2H, s, axle CH_2), 4.85 (2H, s, axle CH_2), 4.34 - 4.47 (2H, m, axle CH_2), 4.06 - 4.13 (2H, m, axle CH_2), 3.92 (3H, s, triazolium CH_3), 3.51 - 4.27 (20H, m, macrocycle CH_2), 2.20 - 2.32 (2H, m, axle CH_2), 1.29 (54H, s, stopper CH_3)

^{19}**F NMR** (470 MHz, $CDCl_3$) δ = -72.4 (d, ^1J = 708 Hz, PF_6).

^{31}**P NMR** (202 MHz, $CDCl_3$) δ = -144.3 (sept, ^1J = 709 Hz, PF_6).

Two station XB rotaxane 5·A

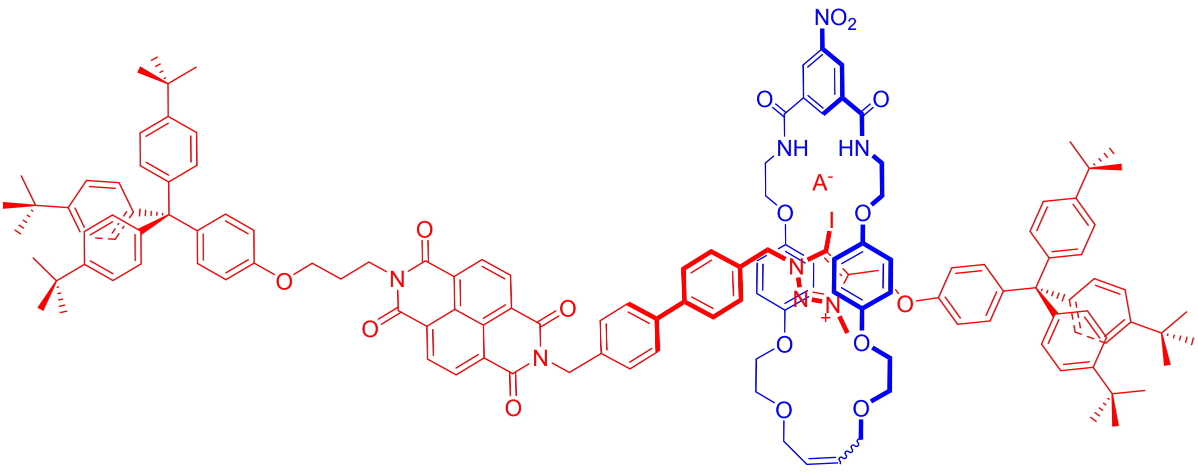

5·I

^{1}**H NMR** (400MHz, 4:1 $CDCl_3$:MeOD) δ = 9.25 (1H, s, internal macrocycle Ar*H*), 8.85 (2H, s, external macrocycle Ar*H*), 8.63 - 8.77 (4H, m, NDI Ar*H*), 8.38 - 8.46 (2H, m, macrocycle N*H*), 7.38 - 7.64 (8H, m, biphenyl Ar*H*), 6.90 - 7.30 (30H, m, stopper Ar*H*), 6.56 - 6.64 (2H, m, stopper Ar*H*), 6.19 - 6.29 (8H, m, hydroquinone macrocycle Ar*H*), 5.43 - 5.52 (2H, m, alkene macrocycle C*H*), 5.37 (2H, s, axle CH_2), 4.99 (2H, s, axle CH_2), 4.57 (2H, s, axle CH_2), 4.31 -

4.49 (6H, m, macrocycle and axle C*H*$_2$), 4.15 (3H, s, triazolium C*H*$_3$), 4.02 - 4.11 (2H, m, axle C*H*$_2$), 3.44 - 3.97 (16H, m, macrocycle C*H*$_2$), 2.16 - 2.27 (2H, m, axle C*H*$_2$), 1.25 (54H, s, stopper C*H*$_3$)

5·PF$_6$

^{1}H NMR (500MHz, CDCl$_3$) δ = 8.97 (3H, br. s, external and internal macrocycle Ar*H*), 8.57 - 8.74 (4H, m, NDI Ar*H*), 7.43 - 7.62 (8H, m, biphenyl Ar*H*), 7.01 - 7.32 (28H, m, stopper Ar*H*), 6.84 - 6.94 (2H, m, stopper Ar*H*), 6.72 (2H, d, *J* = 8.9 Hz, stopper Ar*H*), 5.98 (10H, br. s., hydroquinone macrocycle Ar*H* and alkene macrocycle C*H*), 5.43 (2H, br. s, axle C*H*$_2$), 5.30 (2H, br. s., axle C*H*$_2$), 5.00 (2H, br. s, axle C*H*$_2$), 4.34 - 4.43 (2H, m, axle C*H*$_2$), 4.31 (3H, s, triazolium C*H*$_3$), 3.57 - 4.13 (22H, m, axle and macrocycle C*H*$_2$), 2.21 - 2.29 (2H, m, axle C*H*$_2$), 1.29 (54H, s, stopper C*H*$_3$)

^{19}F NMR (470 MHz, CDCl$_3$) δ = -72.5 (d, ^1J = 707 Hz, PF$_6$).

^{31}P NMR (202 MHz, CDCl$_3$) δ = -144.3 (sept, ^1J = 709 Hz, PF$_6$).

One station HB rotaxane 6·A

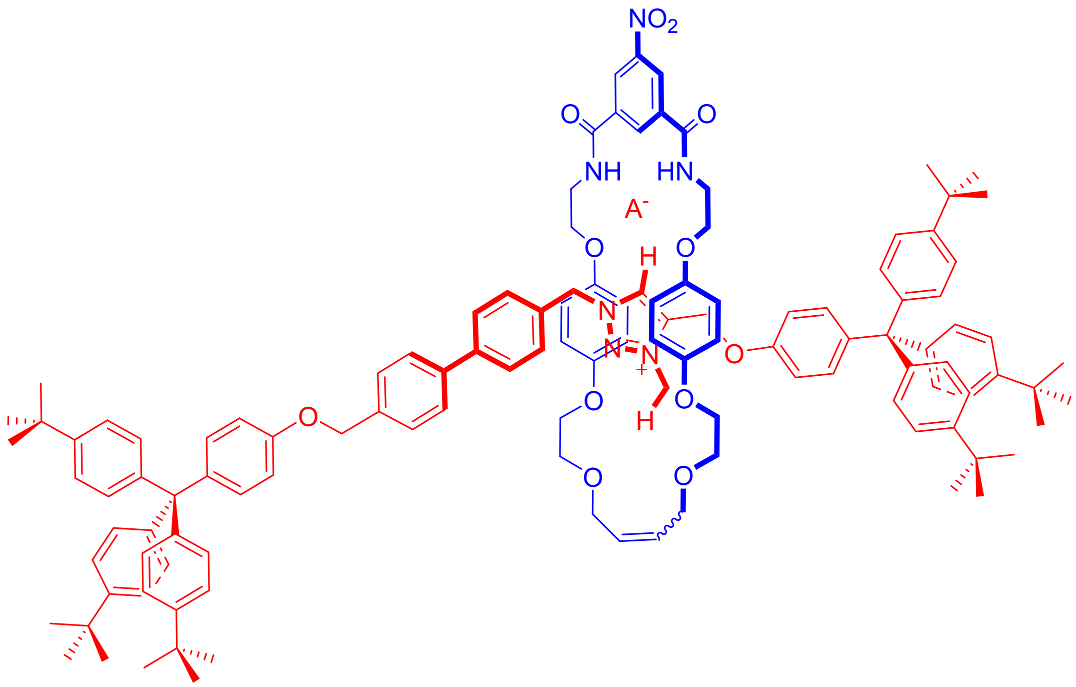

6·I

^{1}H NMR (400MHz, 4:1 CDCl$_3$:MeOD) δ = 9.22 (1H, s, internal macrocycle Ar*H*), 8.73 (3H, s, triazolium and external macrocycle Ar*H*), 8.62 - 8.70 (2H, m, macrocycle N*H*), 7.36 - 7.53 (8H, m, biphenyl Ar*H*), 6.94 - 7.29 (30H, m, stopper Ar*H*), 6.84 (2H, d, *J* = 8.9 Hz, stopper Ar*H*), 6.24 - 6.60 (8H, m, hydroquinone macrocycle Ar*H*), 5.60 - 5.70 (2H, m, alkene macrocycle C*H*), 5.31 (2H, s, axle C*H*$_2$), 5.04 (2H, s, axle C*H*$_2$), 4.80 (2H, s, axle C*H*$_2$), 4.67 - 4.78 (2H, m, macrocycle C*H*$_2$), 4.29 - 4.39 (2H, m, macrocycle C*H*$_2$), 3.85 (3H, s, triazolium C*H*$_3$), 3.54 - 4.07 (16H, m, macrocycle C*H*$_2$), 1.24 (54H, s, stopper C*H*$_3$)

6·PF_6

^{1}H NMR (500MHz, $CDCl_3$) δ = 8.87 (2H, s, external macrocycle Ar*H*), 8.50 (1H, br. s, internal macrocycle Ar*H*), 8.25 (1H, br. s, triazolium Ar*H*), 7.43 - 7.60 (8H, m, biphenyl Ar*H*), 7.03 - 7.29 (28H, m, stopper Ar*H*), 6.95 (2H, d, *J* = 8.5 Hz, stopper Ar*H*), 6.88 (2H, d, *J* = 8.7 Hz, stopper Ar*H*), 6.26 - 6.58 (8H, m, hydroquinone macrocycle Ar*H*), 5.64 - 5.73 (2H, m, alkene macrocycle C*H*), 5.21 (2H, s, axle CH_2), 5.07 (2H, s, axle CH_2), 4.73 (2H, s, axle CH_2), 4.33 - 4.51 (4H, m, macrocycle CH_2), 3.79 (3H, s, triazolium CH_3), 3.52 - 4.15 (16H, m, macrocycle CH_2), 1.31 (54H, s, stopper CH_3)

^{19}F NMR (470 MHz, $CDCl_3$) δ = -72.7 (d, ^1J = 706 Hz, PF_6).

^{31}P NMR (202 MHz, $CDCl_3$) δ = -144.3 (sept, ^1J = 709 Hz, PF_6).

One station XB rotaxane 7·A

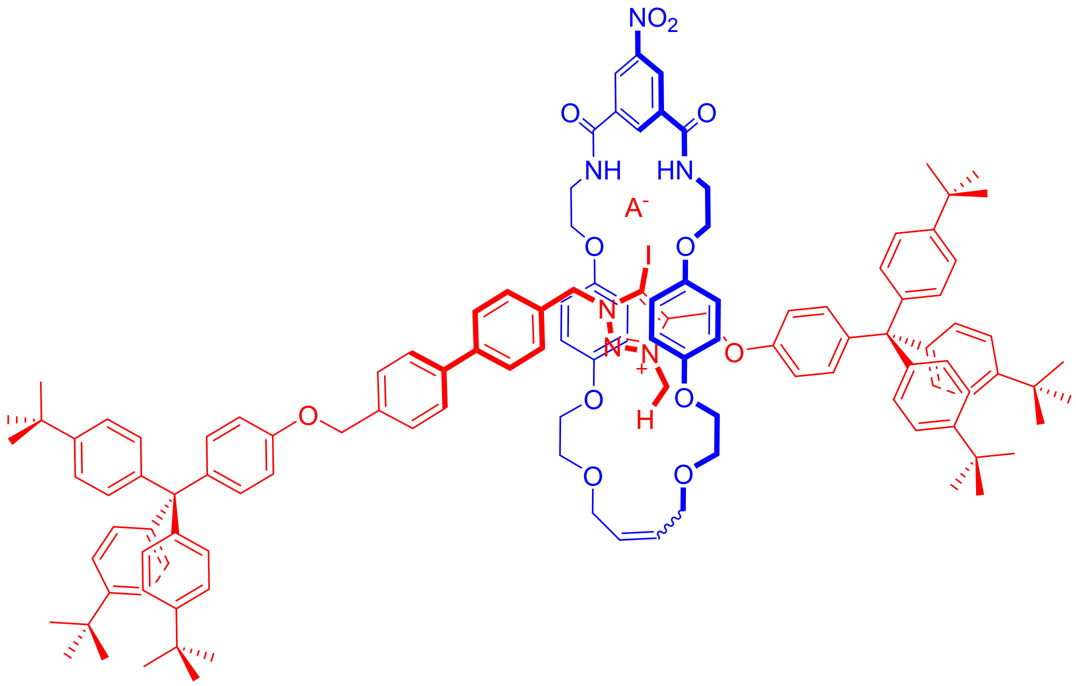

7·I

^{1}H NMR (400MHz, 4:1 $CDCl_3$:MeOD) δ = 9.28 (1H, s, internal macrocycle Ar*H*), 8.88 (2H, d, *J* = 1.3 Hz, external macrocycle Ar*H*), 7.42 - 7.66 (8H, m, biphenyl Ar*H*), 6.90 - 7.32 (30H, m, stopper Ar*H*), 6.80 - 6.87 (2H, m, stopper Ar*H*), 6.29 - 6.43 (8H, m, hydroquinone macrocycle Ar*H*), 5.34 - 5.42 (2H, m, alkene macrocycle C*H*), 5.06 (2H, s, axle CH_2), 4.92 (2H, s, axle CH_2), 4.50 (2H, s, axle CH_2), 4.43 - 4.62 (4H, m, macrocycle CH_2), 4.13 (3H, s, triazolium CH_3), 3.94 - 4.16 (4H, m, macrocycle CH_2), 3.45 - 3.90 (12H, m, macrocycle CH_2), 1.25 (54H, s, stopper CH_3)

7·PF₆

¹H NMR (400MHz, $CDCl_3$) δ = 8.83 - 8.92 (3H, m, internal and external macrocycle Ar*H*), 7.38 - 7.59 (8H, m, biphenyl Ar*H*), 7.01 - 7.32 (28H, m, stopper Ar*H*), 6.90 - 6.98 (2H, m, stopper Ar*H*), 6.80 - 6.90 (2H, m, stopper Ar*H*), 6.31 - 6.56 (8H, m, hydroquinone macrocycle Ar*H*), 5.49 - 5.58 (2H, m, alkene macrocycle C*H*), 5.03 (4H, s, axle CH_2), 4.72 (2H, br. s, axle CH_2), 4.34 - 4.52 (2H, m, macrocycle CH_2), 4.18 - 4.32 (2H, m, macrocycle CH_2), 4.15 (3H, s, triazolium CH_3), 3.52 - 4.12 (16H, m, macrocycle CH_2), 1.30 (54H, s, stopper CH_3)

^{19}F NMR (470 MHz, $CDCl_3$) δ = -72.5 (d, ^1J = 706 Hz, PF_6).

^{31}P NMR (202 MHz, $CDCl_3$) δ = -144.3 (sept, ^1J = 709 Hz, PF_6)

Method for Estimation of Percentage Occupancies

A Δδ scale in each anionic state of the system (counter anion) for each station (NDI and triazolium) was constructed using the δ values of the station's diagnostic protons, $H_{e,e'}$ and H_l respectively, obtained from their ¹H NMR spectra. The following equation was then used to estimate the occupancy of a station:

Occ.(station) = Δδ(rotaxane)/Δδ(model)

Where,

Δδ(rotaxane) = δ(0% occ.) – δ(rotaxane)

Δδ(model) = δ(0% occ.) − δ(100% occ.)

And,

δ(100% occ.) = δ of the station's diagnostic protons in the model one station rotaxane

δ(0% occ.) = δ of the station's diagnostic protons in the two station axle

δ(rotaxane) = δ of the station's diagnostic protons in the two station rotaxane

The estimation of the percentage occupancies of each station in the XB two station rotaxane **5·PF₆** in $CDCl_3$ is used as an example to demonstrate this.

For the triazolium station (Figures S6 to S9):

δ(0% occ.) = δ(H_l) (**2·PF₆**) = 4.41 ppm

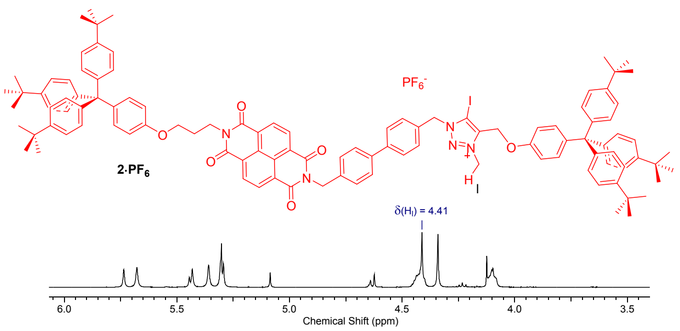

*Figure S6. Truncated ^{1}H NMR spectrum of two station axle **2·PF₆** (CDCl₃, 298 K, 400 MHz)*

δ(100% occ.) = δ(H_l) (**7·PF₆**) = 4.15 ppm

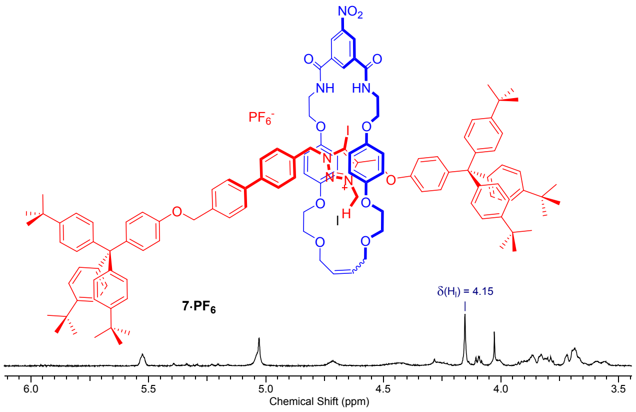

*Figure S7. Truncated ^{1}H NMR spectrum of one station rotaxane **7·PF₆** (CDCl₃, 298 K, 400 MHz)*

δ(rotaxane) = δ(H_l) (**5·PF₆**) = 4.31 ppm

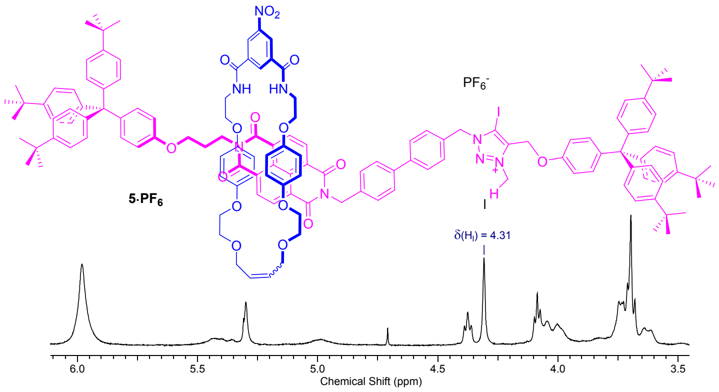

*Figure S8. Truncated ^{1}H NMR spectrum of two station rotaxane **5·PF₆** (CDCl₃, 298 K, 400 MHz)*

Therefore: Occ.(triazolium) = (4.41 – 4.31) / (4.41 – 4.15) = 0.38 = 38%

And this implies: Occ.(NDI) = 1 – 0.38 = 0.62 = 62%

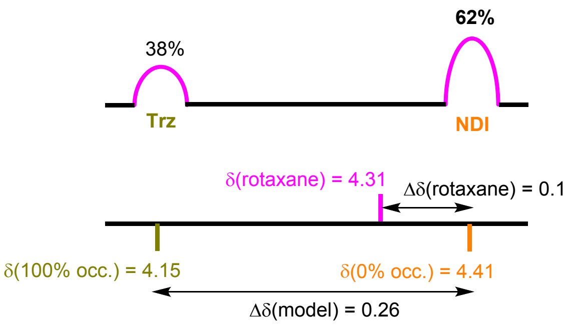

Figure S9. Δδ scale for the triazolium station as the hexafluorphosphate salt in $CDCl_3$

For the NDI station (Figures S10 to S13):

δ(0% occ.) = δ($H_{e,e'}$) (**2·PF₆**) = 8.76 ppm

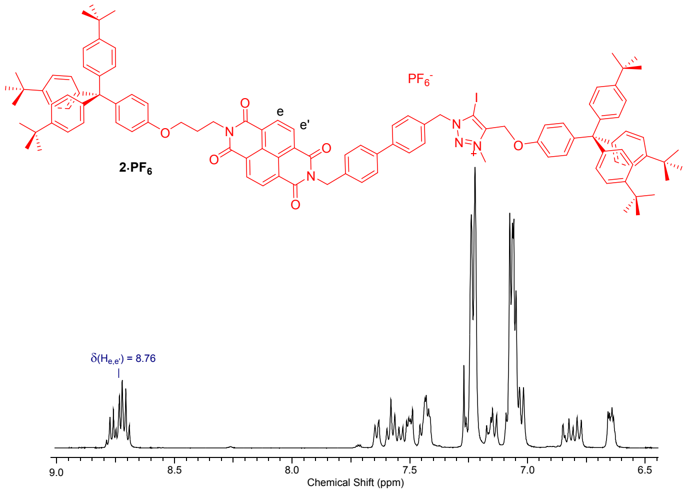

*Figure S10. Truncated ¹H NMR spectrum of two station axle **2·PF₆** ($CDCl_3$, 298 K, 400 MHz)*

δ(100% occ.) = δ($H_{e,e'}$) (**8**) = 8.56 ppm

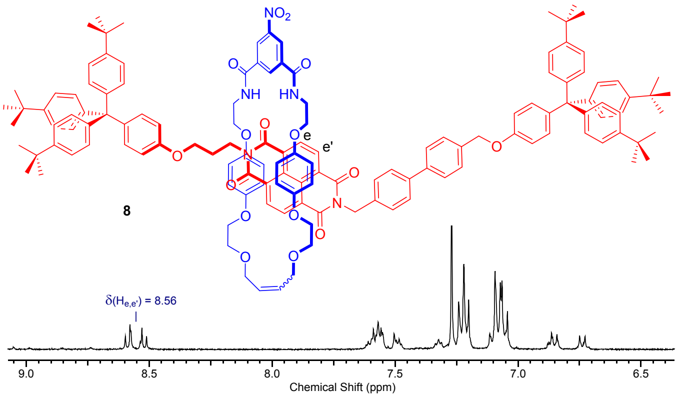

*Figure S11. Truncated ¹H NMR spectrum of one station rotaxane **8** ($CDCl_3$, 298 K, 400 MHz)*

δ(rotaxane) = δ($H_{e,e'}$) (**5·PF$_6$**) = 8.65 ppm

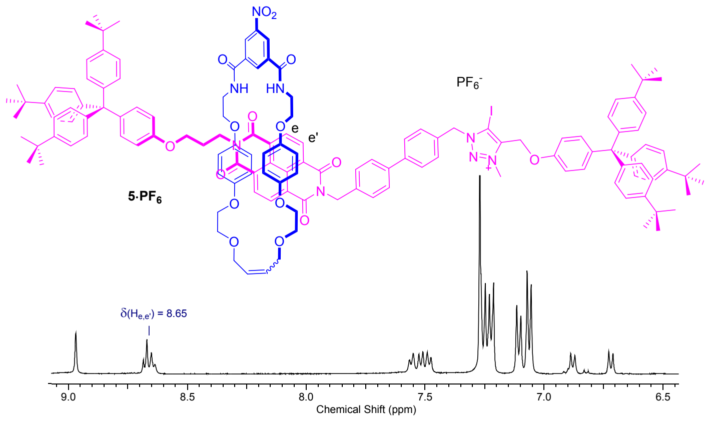

Figure S12. Truncated ^{1}H NMR spectrum of two station rotaxane ***5·PF$_6$*** *($CDCl_3$, 298 K, 400 MHz)*

Occ.(NDI) = (8.76 – 8.65) / (8.76 – 8.56) = 0.55 = 55%

And this implies: Occ.(triazolium) = 1 – 0.55 = 0.45 = 45%

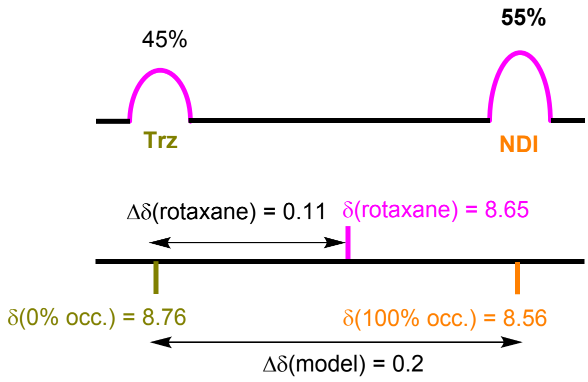

Figure S13. Δδ scale for the NDI station as the hexafluorphosphate salt in $CDCl_3$

Definition data used to estimate the % occupancies reported in the main article (Figures 7, 8 and 9) were taken only from the triazolium shifts, δ(H_l), since the NDI protons $H_{e,e'}$ often produced a complex multiplet (see **2·PF$_6$**) and were frequently found to overlap with the peak from the macrocycle protons H_α (see **4·Cl**), both of which made the diagnostic shift of δ($H_{e,e'}$) more difficult to determine. In addition Δδ(model) for NDI was always found to be smaller than Δδ(model) for the triazolium station.

^{1}H NMR spectra of the compounds **1·Cl**, **1·I**, **1·PF$_6$**, **2·Cl**, **2·I**, **2·PF$_6$**, **4·Cl**, **4·I**, **4·PF$_6$**, **5·Cl**, **5·I**, **5·PF$_6$**, **6·Cl**, **6·I**, **6·PF$_6$**, **7·Cl**, **7·I**, **7·PF$_6$** and **8** in $CDCl_3$ gave the following δ(H_l) and δ($H_{e,e'}$) values that were then used to estimate the percentage occupancies of the two station rotaxanes **4·A** and **5·A** shown in Figure 7 in the main article (Tables S1 and S2).

*Table S1. Tabulated δ(H_l) and δ($H_{e,e'}$) values that were used to estimate the percentage occupancies of the two station HB rotaxane **4·A** in $CDCl_3$*

Station	Anion	δ(rotaxane)	δ(0% occ.)	δ(100% occ.)	% Occ.(triazolium)	% Occ.(NDI)
	Cl^-	3.88	4.38	3.88	100	0
**Triazolium**	I^-	3.87	4.41	3.87	100	0
**δ(H_l)**	PF_6^-	3.92	4.33	3.79	76	24
	Cl^-	8.76			100	0
**NDI**	I^-	8.76	8.76	8.56	100	0
**δ($H_{e,e'}$)**	PF_6^-	8.73			85	15

*Table S2. Tabulated δ(H_l) and δ($H_{e,e'}$) values that were used to estimate the percentage occupancies of the two station XB rotaxane **5·A** in $CDCl_3$*

Station	Anion	δ(rotaxane)	δ(0% occ.)	δ(100% occ.)	% Occ. (triazolium)	% Occ. (NDI)
	Cl^-	4.18	4.40	4.16	92	8
**Triazolium**	I^-	4.16	4.37	4.15	95	5
**δ(H_l)**	PF_6^-	4.31	4.41	4.15	38	62
	Cl^-	8.76			100	0
**NDI**	I^-	8.76	8.76	8.56	100	0
**δ($H_{e,e'}$)**	PF_6^-	8.65			45	55

^{1}H NMR spectra of the compounds **1·Cl**, **1·I**, **1·PF$_6$**, **2·Cl**, **2·I**, **2·PF$_6$**, **4·Cl**, **4·I**, **4·PF$_6$**, **5·Cl**, **5·I**, **5·PF$_6$**, **6·Cl**, **6·I**, **6·PF$_6$**, **7·Cl**, **7·I**, **7·PF$_6$** and **8** in 4:1 $CDCl_3$:MeOD gave the following δ(H_l) and δ($H_{e,e'}$) values that were then used to estimate the percentage occupancies of the two station rotaxanes **4·A** and **5·A** shown in Figure 8 in the main article (Tables S3 and S4).

*Table S3. Tabulated δ(H_l) and δ($H_{e,e'}$) values that were used to estimate the percentage occupancies of the two station HB rotaxane **4·A** in 4:1 $CDCl_3$:CD_3OD*

Station	Anion	δ(rotaxane)	δ(0% occ.)	δ(100% occ.)	% Occ.(triazolium)	% Occ.(NDI)
	Cl^-	3.92	4.33	3.86	87	13
**Triazolium**	I^-	4.00	4.35	3.85	70	30
**δ(H_l)**	PF_6^-	4.01	4.31	3.86	67	33
	Cl^-	8.69			95	5
**NDI**	I^-	8.67	8.70	8.51	84	16
**δ($H_{e,e'}$)**	PF_6^-	8.65			74	26

*Table S4. Tabulated δ(H_l) and δ($H_{e,e'}$) values that were used to estimate the percentage occupancies of the two station XB rotaxane **5·A** in 4:1 $CDCl_3$:CD_3OD*

Station	Anion	δ(rotaxane)	δ(0% occ.)	δ(100% occ.)	% Occ.(triazolium)	% Occ.(NDI)
	Cl^-	4.17	4.42	4.17	100	0
**Triazolium**	I^-	4.15	4.39	4.13	92	8
**δ(H_l)**	PF_6^-	4.25	4.37	4.14	52	48
	Cl^-	8.70			100	0
**NDI**	I^-	8.70	8.70	8.51	100	0
**δ($H_{e,e'}$)**	PF_6^-	8.63			63	37

^{1}H NMR spectra of the compounds **1·Cl**, **1·I**, **1·PF$_6$**, **2·Cl**, **2·I**, **2·PF$_6$**, **4·Cl**, **4·I**, **4·PF$_6$**, **5·Cl**, **5·I**, **5·PF$_6$**, **6·Cl**, **6·I**, **6·PF$_6$**, **7·Cl**, **7·I**, **7·PF$_6$** and **8** in 1:1 $CDCl_3$:MeOD gave the following δ(H_l) and δ($H_{e,e'}$) values that were then used to estimate the percentage occupancies of the two station rotaxanes **4·A** and **5·A** shown in Figure 9 in the main article (Tables S5 and S6).

*Table S5. Tabulated δ(H_l) and δ($H_{e,e'}$) values that were used to estimate the percentage occupancies of the two station HB rotaxane **4·A** in 1:1 $CDCl_3$:CD_3OD*

Station	Anion	δ(rotaxane)	δ(0% occ.)	δ(100% occ.)	% Occ.(triazolium)	% Occ.(NDI)
**Triazolium**	I^-	4.21	4.36	3.94	36	64
**δ(H_l)**	PF_6^-	4.21	4.34	3.95	33	67
	Cl^-	4.17	4.35	3.98	49	51
**NDI**	I^-	8.63			50	50
**δ($H_{e,e'}$)**	PF_6^-	8.61	8.71	8.55	37	63
	Cl^-	8.61			37	63

*Table S6. Tabulated δ(H_l) and δ($H_{e,e'}$) values that were used to estimate the percentage occupancies of the two station XB rotaxane **5·A** in 1:1 $CDCl_3$:CD_3OD*

Station	Anion	δ(rotaxane)	δ(0% occ.)	δ(100% occ.)	% Occ.(triazolium)	% Occ.(NDI)
**Triazolium**	I^-	4.26	4.43	4.16	63	37
**δ(H_l)**	PF_6^-	4.33	4.40	4.19	33	67
	Cl^-	4.30	4.40	4.19	48	52
**NDI**	I^-	8.65	8.71	8.55	60	40
**δ($H_{e,e'}$)**	PF_6^-	8.61			37	63
	Cl^-	8.63			50	50

Part V: X-ray Diffraction

Single crystal X-ray diffraction data for **5·Cl** were collected using synchrotron radiation on Beamline I19 at Diamond Lightsource[8] The diffractometer was equipped with a Cryostream N2 open-flow cooling device,[9] and the data were collected at 100(2) K. Cell parameters and intensity data (including inter-frame scaling) were processed using CrysAlis Pro.[10]

All structures were solved by charge-flipping methods using SUPERFLIP.[11] All structures were refined using full-matrix least-squares on F^2 within the CRYSTALS suite.[12,13] All non-hydrogen atoms were refined with anisotropic displacement parameters. Hydrogen atoms were generally visible in the difference map and their positions and displacement parameters were refined using restraints prior to inclusion into the model using riding constraints.[14]

Discussion of Structures

Crystals suitable for X-ray diffraction structural analysis were obtained for the chloride salt of the XB rotaxane **5·Cl** by slow evaporation of a solution of the rotaxane in 1:1 $CDCl_3$:CD_3OD. All crystals were small and very weakly diffracting; despite the use of synchrotron radiation, diffraction was relatively weak and data were of relatively low quality. Despite this, the structure of the rotaxane, the location of the macrocycle along the two station axle, and the locations of the anion, chloroform and water molecules can be determined unambiguously. The macrocycle can be clearly seen, situated at the iodotriazolium station with the coordinating chloride anion encapsulated within the rotaxane's three-dimensional binding cavity. Short contacts between the I···Cl^- (2.987(6) Å) and N–H···Cl^- (2.626(14) Å) indicate strong halogen and hydrogen bonds, respectively, with distances shorter than the sum of the van der Waals' radii (XB: 80%, HB: 93%), which contribute to the stabilisation of the rotaxane–anion complex.

Areas of diffuse electron density, which appear to result from disordered solvent molecules was present. While one molecule of chloroform and one molecule of water were apparent from the difference map, the remaining diffuse electron density could not be modelled sensibly. Therefore PLATON-SQUEEZE[15,16] was used to include this electron density in the refinement.

Thermal motion of the stopper-^{t}Bu groups and the macrocycle polyether part are evident. It was possible to model this motion as positional disorder over two sites. However in some cases, the ellipsoids for the stopper-^{t}Bu groups are quite large as a result of relatively low quality data. It was necessary to apply restraints to bond lengths and angles, as well as thermal and vibrational ellipsoid parameters of the affected atoms to ensure a chemically sensible refinement.

Accommodating the chloride anion within the rotaxane cavity appears to be quite sterically demanding, causing the polyether-alkene part of the macrocycle to adopt a relatively strained geometry. This appears to force a hydrogen atom from the alkene group and a hydrogen atom from the iodotriazolium-methyl group close together.

Hydrogen atoms were located from the difference map but those attached to carbon atoms were repositioned geometrically. The H atoms were initially refined with soft restraints on the bond lengths and angles to regularise their geometry and U_{iso}(H), after which the positions were refined with riding constraints.[14] Water treatment: the H atoms were located in the difference map near the oxygen atom and refined with soft restraints, whereafter the positions were refined with riding constraints.

References

1. B.-Y. Lee, S. R. Park, H. B. Jeon and K. S. Kim, *Tetrahedron Lett.*, 2006, **47**, 5105–5109.
2. P. Pengo, G. D. Pantoş, S. Otto and J. K. M. Sanders, *J. Org. Chem.*, 2006, **71**, 7063–7066.
3. M. R. Sambrook, P. D. Beer, M. D. Lankshear, R. F. Ludlow and J. A. Wisner, *Org. Biomol. Chem.*, 2006, **4**, 1529–1538.
4. L. M. Hancock and P. D. Beer, *Chem. Commun.*, 2011, **47**, 6012–6014.
5. V. Aucagne, K. D. Hänni, D. A. Leigh, P. J. Lusby and D. B. Walker, *J. Am. Chem. Soc.*, 2006, **128**, 2186–2187.
6. H. Zheng, W. Zhou, J. Lv, X. Yin, Y. Li, H. Liu and Y. Li, *Chem. – Eur. J.*, 2009, **15**, 13253–13262.
7. H. W. Gibson, S. H. Lee, P. T. Engen, P. Lecavalier, J. Sze, Y. X. Shen and M. Bheda, *J. Org. Chem.*, 1993, **58**, 3748–3756.
8. Nowell, H.; Barnett, S. A.; Christensen, K. E.; Teat, S. J.; Allan, D. R. *J. Synchrotron Rad.* **2012**, *19*, 435- 441.
9. Cosier, J.; Glazer, A. M. *J. Appl. Crystallogr.* **1986**, *19*, 105-107.
10. CrysAlis Pro, Agilent Technologies, 2010.
11. Palatinus, L.; Chapuis, G. *J. Appl. Crystallogr.* **2007**, *40*, 786-790.
12. Betteridge, P. W.; Carruthers, J. R.; Cooper, R. I.; Prout, K.; Watkin, D. J. *J. Appl. Crystallogr.* **2003**, *36*, 1487.
13. Parois, P.; Cooper, R. I.; Thompson, A. L. *Chem. Cent. J*. 2015, **9**, 30.
14. Cooper, R. I.; Thompson, A. L.; Watkin, D. J. *J. Appl. Crystallogr.* **2010**, *43*, 1100-1107.
15. Spek, A. *J. Appl. Crystallogr.* **2003**, *36*, 7-13.
16. van der Sluis, P.; Spek, A. L. *Acta Crystallogr., Sect. A: Found. Crystallogr.* **1990**, *46*, 194-201.
